# Cellular and Genomic Features of Muscle Differentiation from Isogenic Fibroblasts and Myoblasts

**DOI:** 10.3390/cells12151995

**Published:** 2023-08-03

**Authors:** Louise Benarroch, Julia Madsen-Østerbye, Mohamed Abdelhalim, Kamel Mamchaoui, Jessica Ohana, Anne Bigot, Vincent Mouly, Gisèle Bonne, Anne T. Bertrand, Philippe Collas

**Affiliations:** 1 Sorbonne Université, Inserm, Institut de Myologie, Centre de Recherche en Myologie, 75013 Paris, France; louise.benarroch@inserm.fr (L.B.); k.mamchaoui@institut-myologie.org (K.M.); j.ohana@institut-myologie.org (J.O.); anne.bigot@sorbonne-universite.fr (A.B.); vincent.mouly@upmc.fr (V.M.); gisele.bonne@inserm.fr (G.B.); 2 Department of Molecular Medicine, Institute of Basic Medical Sciences, Faculty of Medicine, University of Oslo, 0372 Oslo, Norway; j.k.madsen-osterbye@medisin.uio.no (J.M.-Ø.); m.i.m.abdelhalim@medisin.uio.no (M.A.); 3 Department of Immunology and Transfusion Medicine, Oslo University Hospital, 0372 Oslo, Norway

**Keywords:** chromatin, fibroblast, lamina-associated domain, myotube, myogenic conversion, myogenesis, transcriptome

## Abstract

The ability to recapitulate muscle differentiation in vitro enables the exploration of mechanisms underlying myogenesis and muscle diseases. However, obtaining myoblasts from patients with neuromuscular diseases or from healthy subjects poses ethical and procedural challenges that limit such investigations. An alternative consists in converting skin fibroblasts into myogenic cells by forcing the expression of the myogenic regulator MYOD. Here, we directly compared cellular phenotype, transcriptome, and nuclear lamina-associated domains (LADs) in myo-converted human fibroblasts and myotubes differentiated from myoblasts. We used isogenic cells from a 16-year-old donor, ruling out, for the first time to our knowledge, genetic factors as a source of variations between the two myogenic models. We show that myo-conversion of fibroblasts upregulates genes controlling myogenic pathways leading to multinucleated cells expressing muscle cell markers. However, myotubes are more advanced in myogenesis than myo-converted fibroblasts at the phenotypic and transcriptomic levels. While most LADs are shared between the two cell types, each also displays unique domains of lamin A/C interactions. Furthermore, myotube-specific LADs are more gene-rich and less heterochromatic than shared LADs or LADs unique to myo-converted fibroblasts, and they uniquely sequester developmental genes. Thus, myo-converted fibroblasts and myotubes retain cell type-specific features of radial and functional genome organization. Our results favor a view of myo-converted fibroblasts as a practical model to investigate the phenotypic and genomic properties of muscle cell differentiation in normal and pathological contexts, but also highlight current limitations in using fibroblasts as a source of myogenic cells.

## 1. Introduction

Myogenesis is a highly regulated process underlying muscle development and regeneration [1]. Muscle fibers are post-mitotic multinucleated cells generated through the fusion of myoblasts derived from muscle precursor cells. Muscle precursor cells are responsible for the growth and repair of muscle fibers; they are quiescent and express the paired-homeobox transcription factor PAX7 [2]. Upon muscle growth or regeneration, they exit quiescence, become activated, and proliferate. All activated cells initiate expression of the myogenic regulator factors MYOD and MYF5, both involved in myogenic commitment [2]; however, whereas a minority returns to quiescence to replenish the progenitor pool, others engage in differentiation. These cells, called myoblasts, can proliferate and, upon extracellular cues, exit the cell cycle, downregulate PAX7, and express myogenin (MYOG) [2], another regulator factor involved in myoblast engagement into myogenic differentiation. Differentiated cells, called myocytes, fuse to form multinucleated myofibers [3].

Being able to recapitulate myogenesis in vitro has proven invaluable in investigating mechanisms of muscle differentiation and muscle pathologies [4]. In vitro muscle differentiation recapitulates myoblast fusion to form multinucleated cells named myotubes [4]. However, obtaining myoblasts from patients with neuromuscular disorders depends on availability and raises ethical issues that limit such investigations.

As an alternative, myogenic cellular models have been developed, among which are myogenic cells derived from skin fibroblasts [5,6]. MYOD is the first transcription factor identified that, when expressed ectopically, is able to elicit a myogenic gene expression program [7,8]. Human fibroblasts can be immortalized by transduction of telomerase [9], and myo-converted by induced expression of the *Myod* gene. This forces the fibroblasts to withdraw from the cell cycle, express muscle-specific markers and fuse into multinucleated cells resembling myotubes differentiated from myoblasts in vitro [6]. This system has been used to investigate pathophysiological mechanisms [10,11,12,13] or therapeutic strategies [5,14,15] in neuromuscular disorders. However, rigorous comparisons of phenotype, transcriptome and genome organization on an isogenic background in myogenic cells derived from *Myod*-induced fibroblasts or from muscle biopsy-derived myoblasts have not been reported.

An important regulator of gene expression during differentiation is the association of chromatin with the nuclear lamina at the periphery of the nucleus [16]. The nuclear lamina is a meshwork of A-type lamins (the lamins A and C splice variants) and B-type lamins (lamins B1 and B2) [17]. A- and B-type lamins associate with chromatin via lamina-associated domains (LADs), regions that are typically gene-poor, enriched in heterochromatin marked by di- and tri-methylated histone H3 lysine K9 (H3K9me2/me3) and containing mostly repressed genes [18]. While most LADs are conserved between cell types [19,20], others differ by being repositioned, for example, during differentiation [21,22,23,24,25].

Alterations in LADs have been linked to diseases such as cancer [26] or laminopathies [18]. Laminopathies include a wide spectrum of rare diseases, including neuromuscular disorders, mainly caused by dominant mutations in the *LMNA* gene [27,28]. In this pathological context, *LMNA* mutations have been associated with defects in LAD organization and gene expression at the nuclear periphery [11,29,30,31,32,33,34,35,36]. These observations highlight the importance of LADs as genome organizers.

Here, we compare phenotypic, transcriptomic, and LAD features in myo-converted fibroblasts and in myotubes differentiated from myoblasts, in an isogenic context. Our results favor a view of myo-converted fibroblasts as a practical model to investigate the cellular and genomic properties of cells from patients with muscle pathologies, but also point to current limitations in using this model.

## 2. Materials and Methods

### 2.1. Myogenic Induction of Myoblasts and Fibroblasts

Fibroblasts and myoblasts from a 16-year-old individual were derived by the MyoLine cell culture platform of the Center of Research in Myology (https://recherche-myologie.fr/technologies/myoline/; accessed on 29 June 2023) from skin or muscle biopsies from the same individual obtained anonymously from MyoBank (Authorization to distribute human samples ref. AC-2019–3502). Myoblasts and fibroblasts were derived from a paravertebral muscle biopsy and a dorsal skin biopsy, respectively, both carried out at the same time. Myoblasts and fibroblasts were immortalized by transduction of human telomerase (hTERT) (fibroblasts) [5] or hTERT and CDK4 (myoblasts) [37]. Of note, an earlier transcriptomic study comparing primary and immortalized myoblasts and myotubes did not reveal any significant transcriptional alterations [38].

Immortalized myoblasts were cultured in DMEM supplemented with 16% M199 (Invitrogen, Waltham, MA, USA), 4.5 g/L glucose, 20% fetal bovine serum, 0.1% Penicillin-Streptomycin, 5 µg/mL insulin, 0.2 µg/mL dexamethasone (Sigma-Aldrich; Burlington, MA, USA), 25 µg/mL fetuin, 5 ng/mL human epidermal growth factor, and 0.5 ng/mL basic fibroblast growth factor (Life Technologies; Carlsbad, CA, USA). For differentiation, cells were plated on dishes coated with 1% Matrigel (Sigma-Aldrich) at high density in DMEM with 10 µg/mL insulin and 50 µg/mL Penicillin-Streptomycin. After 5 days, myotubes were harvested for assays.

For myo-conversion, immortalized fibroblasts were transduced with a Tet-on inducible lentiviral vector encoding mouse *Myod* [5]. Cells were cultured in DMEM with 4.5 g/L glucose, 10% fetal bovine serum, and 0.1% Penicillin-Streptomycin. For differentiation, cells were then plated on dishes coated with 1% Matrigel at high density and cultured in DMEM with 10 µg/mL insulin, 4 µg/mL doxycycline, and 50 µg/mL Penicillin-Streptomycin. After 5 days, unless otherwise noted, myo-converted fibroblasts were harvested for assays.

All experiments were completed in three independent replicates, each from a separate cell batch.

### 2.2. Immunofluorescence

Cells were differentiated for 5 days on 4-well plates with Nuclon Delta Surface (Thermo Fisher Scientific; Waltham, MA, USA). Cells were fixed for 10 min in 4% paraformaldehyde, permeabilized for 6 min in PBS/0.5% TX-100 and blocked for 30 min with 5% BSA. Primary antibodies (anti-myosin heavy chain (MHC) MF20, DSHB, 1:100; anti-vimentin (D21H3) Cell Signaling No. 5741, 1:100) and secondary antibodies (Alexa Fluor™ 488 goat anti-mouse IgG (H + L), Thermo Fisher Scientific A-11001, 1:500; Alexa Fluor™ 568 goat anti-rabbit IgG (H + L), Thermo Fisher Scientific A-11011, 1:500) were incubated for 45 and 30 min, respectively, in 5% BSA. Slides were mounted in Vectashield/DAPI (Vector Laboratories). Images were taken on a Nikon Ti-2 microscope at 100× or on a Zeiss Axio Observer Apotome at 20× and analyzed with ImageJ (https://imagej.nih.gov/ij/download.html; accessed on 29 June 2023). The fusion index was calculated as the ratio of the number of nuclei per MHC-positive cell over the total number of nuclei per field. Five random fields were analyzed per differentiation, in three differentiation replicates. A total of 15 images were taken per cell type, with more than 100 nuclei per image. Statistics were acquired using GraphPad Prism v.8.3.0.

### 2.3. RNA-Sequencing and RNA-Seq Analysis

Total RNA was extracted with TRIzol (Thermo Fisher Scientific), eluted in 20 µL RNase-free water, and its concentration was estimated by A260. RNA integrity was assessed on an Agilent 2100 Bioanalyzer using the RNA 6000 Nano kit. RNA-seq libraries were prepared using the KAPA mRNA HyperPrep kit (Roche; Basel, Switzerland), and 100 bp paired-end sequencing was completed on a NovaSeq 6000 (Illumina; San Diego, CA, USA).

RNA-seq reads were filtered to remove low-quality reads using fastp (v 0.23.2) [39]. Transcripts were quantified using Salmon package 36 v1.7.0 [40] and Ensembl GRCh38.p13 release 108 [41]. Transcript abundance values were imported into R and summarized with tximport v1.28.0 [42]. DESeq2 v1.36.0 [43], as implemented in SARTools [44], was used to normalize raw counts and apply analyses. Transcripts per million (TPM) were calculated for each transcript. Heatmaps were generated by Ward’s clustering of expression z-scores in R (https://biocorecrg.github.io/CRG_RIntroduction/heatmap-2-function-from-gplots-package.html; accessed on 21 July 2023). Gene Set Enrichment Analysis (GSEA) was completed on normalized read counts [45]. Gene ranking was generated for each comparison with Pearson correlation metrics and analyzed against the mSigDB Hallmarks v7.5.1 gene sets [46].

### 2.4. Chromatin Immunoprecipitation (ChIP)-Sequencing and ChIP-Seq Analysis

Lamin A/C ChIP-seq was completed as described [22,25] from myotubes and myo-converted fibroblasts harvested on day 5 from triplicate differentiations. Cells were fixed in 1% formaldehyde, lysed in 50 mM Tris-HCl, pH 8, 10 mM EDTA, 1% SDS, protease inhibitors, and Na-butyrate, and sonicated into ~200 bp fragments using a Picorupter (Diagenode). After sedimentation, the chromatin supernatant was incubated with anti-lamin A/C antibodies (Santa Cruz sc7292x) at 10 µg/5 × 10^6^ cells. Cross-links were reversed and DNA purified. ChIP of H3K9me3 was completed using anti-H3K9me3 antibodies (Diagenode C15410056) at 2.5 µg/10^6^ cells [22]. ChIP libraries were prepared using a Microplex kit (Diagenode) and sequenced (150 bp paired-end) on a NovaSeq 6000 (Illumina).

ChIP and input sequence reads were mapped to hg38 with Bowtie2 v2.4.1 (https://github.com/BenLangmead/bowtie2; accessed on 21 July 2023) [47]. after removing duplicates using Picard MarkDuplicates (http://broadinstitute.github.io/picard/; accessed on 21 July 2023) and the data were processed as described [25]. Each pair of mapped ChIP and input read files contained the same read depth after down-sampling reads for each chromosome to avoid normalization bias. Lamin A/C and H3K9me3 Log2(ChIP/input) ratios were computed with wiggletools v1.2 (https://github.com/Ensembl/WiggleTools; accessed on 21 July 2023) [48]; bigwig tracks were generated from these ratios in 1 kilobase (kb) bins using bamCompare from Deeptools v3.5.1 (https://github.com/deeptools/deepTools/releases/tag/3.5.1; accessed on 21 July 2023) [49].

For each lamin A/C ChIP replicate, mapped reads were used to call LADs using 10 runs of Enriched Domain Detector (http://github.com/CollasLab/edd; accessed on 1 June 2023) [50] in auto-estimation mode, and mean GapPenalty and BinSize outputs were used for a last EDD run. The final LADs were the union of LADs from the three replicates. Pearson correlations between replicates were computed for LAD overlaps. ChIP-seq data were viewed in the Integrative Genomic Browser (IGV) (www.igv.org; accessed on 1 June 2023) [51].

### 2.5. Intersections between LADs and Genomic Features

Intersects between LADs and genes or H3K9me3 were determined using BEDTools v2.29.2 (https://github.com/arq5x/bedtools2; accessed on 21 July 2023) [52] and BEDOPS v2.4.37 (https://github.com/bedops/bedops/blob/master/CHANGELOG.md; accessed on 21 July 2023) [53]. Intersects required at least one base-pair overlap between features. Gene Ontology Enrichment (GO biological process) was analyzed using Protein ANalysis THrough Evolutionary Relationships (PANTHER) v.14.0 (http://www.pantherdb.org/; accessed on 21 July 2023) [54].

## 3. Results

### 3.1. Myo-Converted Fibroblasts Recapitulate the Phenotypic Characteristics of Myotubes

**Figure 1:**
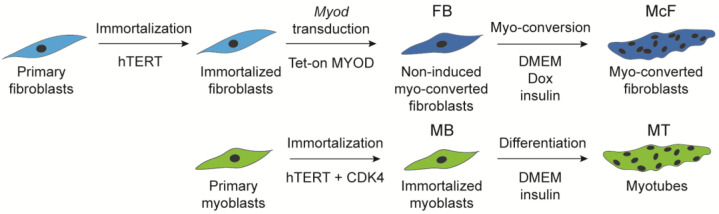
Myogenic differentiation models assessed in this study. Top, primary skin fibroblast (FB) immortalization and induction of *Myod* expression to generate myo-converted fibroblasts (McF). Bottom, immortalization of primary myoblasts (MB) and induction of differentiation to generate myotubes (MT). All cell types examined here are isogenic.

To assess a practical model to investigate phenotypic and genomic aspects of muscle differentiation, we set up two experimental systems (Figure 1): (i) skin fibroblasts from a healthy 16-year-old donor, transduced with inducible *Myod* and stimulated for 5 days with doxycyclin to elicit myogenic conversion; (ii) myoblasts isolated from the same donor and differentiated into myotubes for 5 days. Both fibroblasts and myoblasts were immortalized, which was necessary given the relative scarcity of such dual cell-type donors and to alleviate replicative senescence [55]. Importantly, previous transcriptomic profiling comparing primary and immortalized myoblasts and myotubes has shown no significant transcriptional alterations as a result of immortalization [38].

**Figure 2:**
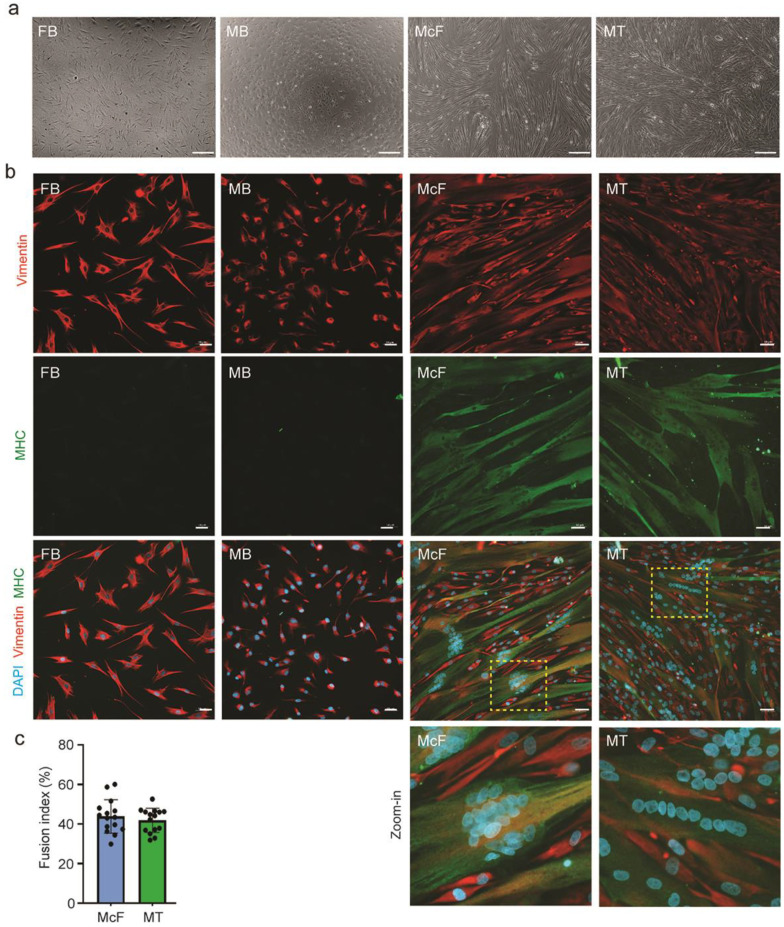
*Myod*-induced fibroblasts differentiate into multinucleated cells. (**a**) Phase contrast microscopy images of FB, MB, McF and MT; bars, 200 µm. (**b**) Immunofluorescence staining of vimentin and MHC; bars, 50 µm. Zoomed-in areas are outlined in yellow. (**c**) Fusion indices of McF and MT (no significant difference; *p* > 0.05, Mann-Whitney test).

Both fibroblasts (from here on, FB) and myoblasts (MB) acquire within 5 days of differentiation an elongated and multinucleated cell morphology (Figure 2a). We refer to differentiated *Myod*-induced fibroblasts as myo-converted fibroblasts (McF) to distinguish them from myotubes (MT) differentiated from myoblasts. Reduced expression of vimentin [56] and enhanced expression of Myosin Heavy Chain (MHC), a myotube and myofiber marker [57], confirm the myogenic phenotype of MT and McF (Figure 2b). Cell fusion indices (ratios of the number of nuclei in MHC-positive cells over the total number of nuclei) are not statistically different (44%) between both models (Figure 2c). We also note that nuclei are more aggregated towards the cell center in McF, in contrast to the central alignment observed in MT (Figure 2b). We conclude so far that myo-converted fibroblasts display a myogenic phenotype, with some features being similar to those of myotubes while nuclear positioning is distinct.

### 3.2. Myod-Induced Fibroblasts Transcriptionally Commit to Myogenesis

**Figure 3:**
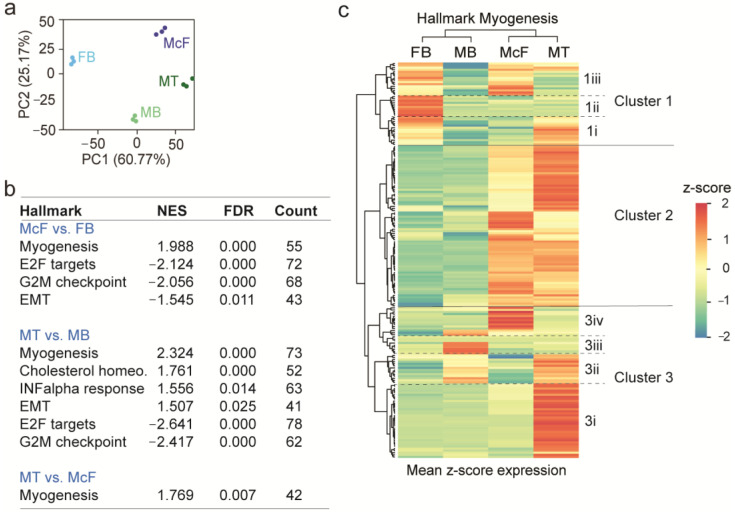
Transcriptome profiling reveals myogenic commitment of *Myod*-induced fibroblasts. (**a**) Principal component analysis of gene expression in FB, MB, McF and MT. Data for each triplicate are shown. (**b**) Enriched Molecular Signature Database Hallmarks in McF vs. FB, MT vs. MB, and MT vs. McF, from GSEA. NES, normalized enrichment score; FDR, false discovery rate; Count, numbers of genes. (**c**) Ward hierarchical clustering of gene expression z-scores across cell types for genes in the hallmark ‘Myogenesis’. Clusters and subclusters addressed in the main text are highlighted.

To gain insights into the ‘FB-McF’ and ‘MB-MT’ models, we characterized by RNA-seq the transcriptome of each cell type. Principal component analysis (PCA) shows that McF and MT are distinct from their undifferentiated counterparts and show proximity along the first principal component axis (PC1); however, they remain distinct along the PC2 axis (Figure 3a). Thus, *Myod* induction elicits in fibroblasts a myogenic gene expression program that resembles, but is not identical to, that of myotubes.

Gene Set Enrichment Analysis (GSEA) reveals the Molecular Signature Database Hallmark ‘Myogenesis’ as the most significant in MT and McF (day 5) relative to MB and FB (FDR = 0.000; Figure 3b), asserting the myogenic commitment of McF. The hallmark ‘Myogenesis’ is also enriched in MT relative to McF (FDR = 0.007), suggesting a more pronounced myogenic fate of the MB-MT model than the FB-McF model. Furthermore, enrichment of the hallmarks ‘E2F targets’ and ‘G2M checkpoint’ in FB and MB relative to McF and MT (FDR = 0.000; Figure 3b) reflects exit from the cell cycle after induction of differentiation. We also note the negative enrichment (which barely reaches significance) of the epithelial-mesenchymal transition (EMT) hallmark (Figure 3b; Figure S1) in McF relative to FB. A number of genes involved in EMT are enriched in the McF system, including interleukins, extracellular matrix components, chemokines and transcription factors. However, the lack of upregulation of other EMT markers in McF (Figure S1) reflects resilience to full mesenchymal commitment of transdifferentiated fibroblasts, in contrast to myotubes (Figure 3b; Figure S1) which stem from myoblasts of already mesenchymal origin.

Clustering gene expression z-scores within the hallmark ‘Myogenesis’ reveals three main clusters (Figure 3c; Figure S2). Cluster 1 reveals (1i) genes upregulated in MT vs. MB but expressed at lower levels in McF, (1ii) genes expressed in FB and downregulated in McF to a level comparable to MT, and (1iii) genes upregulated in FB and McF relative to MB and MT, suggesting partial retention of fibroblast gene expression in McF. Cluster 2 contains genes upregulated in McF and MT and includes genes important for myogenesis and muscle function. Cluster 3 reveals: (3i) genes induced in MT by differentiation; (3ii) genes less expressed in FB and McF than in MB or MT; (3iii) genes expressed in MB and downregulated in MT that retain no/low expression in the FB-McF system; and (3iv) genes upregulated only in McF relative to all other cell types.

**Figure 4:**
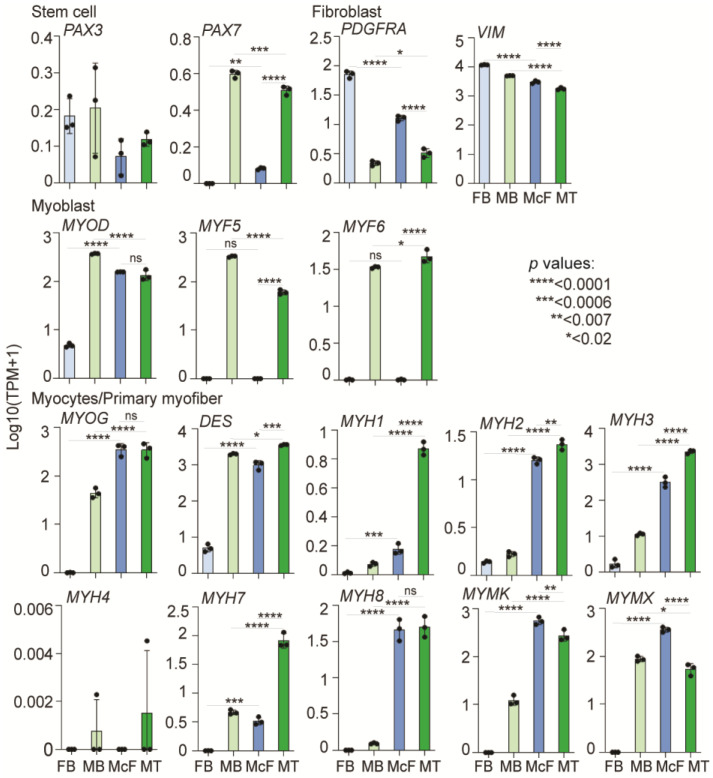
Expression levels of stem cell, fibroblast, myoblast and myotube/myofiber markers in FB, MB, McF and MT. Data are plotted from RNA-seq data for each differentiation triplicate (dots); mean ± SD. *p*-values are shown in the panel; one-way ANOVA.

We then explored changes in expression of fibroblast genes, myogenic regulator factors, and their target genes (Figure 4). The fibroblast markers *PDGFRA* and *VIM* are downregulated in McF compared with FB, the latter in line with protein detection (see Figure 2b) (*p* < 0.0001; one-way ANOVA). *MYF5* is not expressed in McF. This could be explained by the lack of *PAX7* expression (Figure 4) [58], or more likely by the forced induction of MYOD, which, downstream of MYF5, bypasses the need for MYF5 to elicit myogenesis in McF. Consequently, *MYOG*, downstream of MYOD, is expressed in MT and McF. Myocyte and myofiber markers are induced in McF (*p* < 0.007–*p* < 0.0001) to levels comparable to (*MYOG*, *MYH8*, *MYMK*, and *MYMX*) or lower than (*DES*, *MYH1-3*, *MYH7*) those in MT (Figure 4).

We then assessed whether prolonged *Myod* induction with Doxycylin in McF would enable the myogenic gene expression program to extend closer to that of MT. PCA of RNA-seq data after a 7-day induction reveals that McF do not transition closer to MT than day-5 McF; in fact, day-7 McF are more distant from MT than day-5 McF along the PC1 axis (Figure S3a). In day-7 McF, several genes follow an expression profile expected from prolonged myogenic induction (*DES*, *MYOD*1, *MYH4*, *MYMK*, *MYMX*) (Figure S3b). These include myogenin (*MYOG*), which is expected to decrease as it is, in muscle, only transiently expressed early during muscle differentiation to induce expression of the myogenic program [1]. We also note, however, that several other genes do not follow anticipated expression profiles (Figure S3b). Possibly explaining these results, we noted that induction of *Myod* overexpression beyond 7 days elicited detachment of differentiated, multinucleated cells from the culture; thus, we limited induction to 5 days. An account of this observation is given in the Discussion.

Our transcriptome analysis indicates that the McF model supports the induction of a myogenic gene expression program. However, subsets of genes remain expressed at a lower level than in myoblast-derived MT; a minor proportion fails to be downregulated, and others are upregulated only in McF. Thus, although they show myogenic commitment, McF do not engage in a myogenic program to the same extent as myotubes.

### 3.3. Features of Lamin A/C LADs in Myotubes and Myo-Converted Fibroblasts

**Figure 5:**
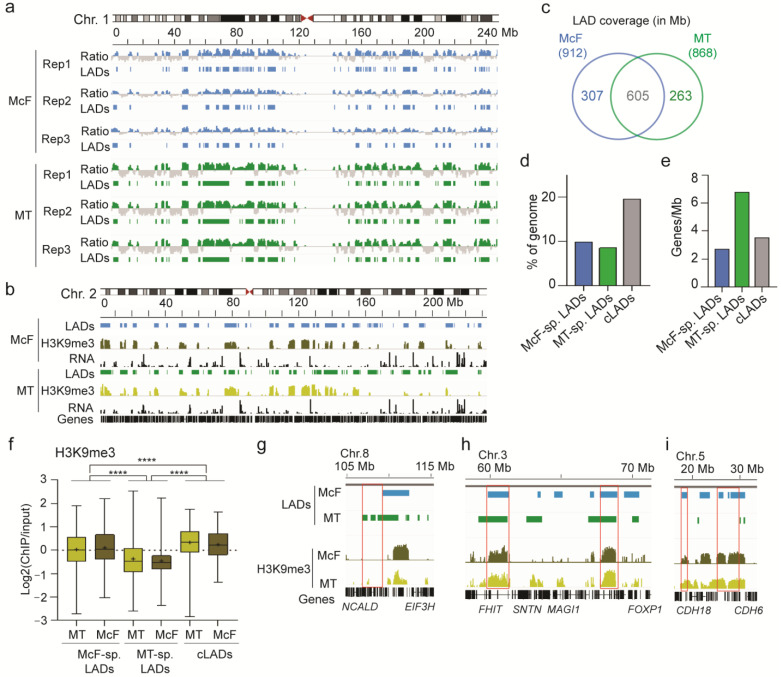
Lamin A/C LADs in myo-converted fibroblasts and myotubes. (**a**) Genome browser view of Log2(lamin A/C ChIP/input) ratios (range −0.4 to +0.4) and LADs on chromosome 1 in McF and MT, for each differentiation replicate (Rep1–3). (**b**) LADs resulting from the union of LADs from each replicate. H3K9me3 enrichment as Log2(H3K9me3 ChIP/input) ratios (range: 0–3) and RNA-seq tracks (counts per million average of the 3 replicates) are shown. (**c**) Venn diagram of LAD genome coverage overlap (in Mb) between McF and MT. Total LAD coverage in McF and MT is 912 and 868 Mb, respectively. (**d**) Genome coverage by McF- and MT-specific LADs and by cLADs, expressed as % of the genome. (**e**) Gene density of McF-specific LADs, MT-specific LADs and cLADs. (**f**) H3K9me3 enrichment in McF-specific LADs, MT-specific LADs and cLADs, for corresponding domains in the other cell type; cross, mean; bar, median; box, 25–75% percentile; whiskers, min-max; **** *p* < 0.0001, ANOVA with Welch’s correction. (**g**–**i**) Genome browser views of (**g**) low H3K9me3 level in MT-specific LADs (box), (**h**) higher H3K9me3 in cLADs (boxes) than in cell type-specific LADs, (**i**) similar H3K9me3 levels in a region of McF-specific LADs (boxes).

To explore the architectural context of both myogenic models, we mapped LADs by ChIP-seq of lamin A/C in MT and McF. For each differentiation replicate, we computed lamin A/C enrichment levels and called them LADs (Figure 5a). Pearson correlations of LAD overlaps between replicates show high reproducibility among the three replicates (Table S1). This allowed us to identify with high confidence, from the union of these LADs, 631 LADs in McF and 739 LADs in MT (Figure 5b; Table S2). Global LAD coverage and mean LAD size are comparable in MT and McF (Figure 5c; Table S2), and most LAD coverage is conserved between the two cell types, with 652 LADs altogether covering 605 Mb (Figure 5b,c); we refer to these shared, or conserved, LADs as cLADs. This is in line with LADs being overall conserved between cell types [20].

To corroborate this view, we assessed the extent of overlap of these McF-MT cLADs with LADs we previously identified by ChIP-seq of lamin A/C in skin fibroblasts from three unrelated donors [30]. We find that 86% of the genome coverage of the McF-MT cLADs (523 Mb out of 605 Mb) are also LADs in these fibroblasts (Figure S4). Thus, the McF-MT cLADs identified here are largely conserved in unrelated skin fibroblasts from unrelated donors. This argues that these cLADs constitute an architectural feature shared between McF and MT. Moreover, 8–10% of the genome is marked as unique LADs specific to McF or MT (Figure 5c,d; Table S2). Thus, these two isogenic myogenic cell types also display differences in radial genome organization, which may reflect their cell type of origin.

Underscoring this view, MT-specific LADs are the most gene-rich (6.8 genes/Mb; Figure 5e), suggesting a weaker heterochromatic feature. Accordingly, levels of H3K9me3, a mark of constitutive heterochromatin, are lower in MT-specific LADs than in cLADs or McF-specific LADs (*p* < 0.0001; ANOVA with Welch’s correction; Figure 5f; exemplified in Figure 5g–i). Weaker H3K9me3 enrichment in MT-specific LADs is also reflected at the level of genes found in these domains (see also Section 3.4), which display depletion of H3K9me3, in contrast to genes in McF-specific and cLADs (Figure S5a). Thus, genes in MT-specific LADs are less heterochromatic than genes localized in McF LADs, and this is despite of a similar H3K9me3 enrichment throughout the genome in both cell types (Figure S5b). In addition, H3K9me3 levels in regions defined as McF-specific LADs or MT-specific LADs are similar in corresponding domains in the other cell type: for example, H3K9me3 levels in McF-specific LADs are similar in the same regions in MT (Figure 5f) despite the fact that the latter are not LADs in MT (Figure 5i); conversely, H3K9me3 levels in MT-specific LADs are similar in the same genomic (non-LAD) regions in McF (Figure 5f,g). Altogether, these findings highlight a weaker heterochromatic state of LADs uniquely identified in MT relative to McF LADs or LADs common to both cell types.

### 3.4. A Subset of Myogenic Genes Is Localized and Expressed in LADs

To appreciate the functionality of MT and McF LADs, we interrogated Gene Ontology (GO) terms enriched for genes uniquely found in these LADs, regardless of their expression. First, we identify 834, 1791, and 2128 protein-coding genes in McF-specific LADs, MT-specific LADs, and cLADs, respectively (Figure S5c; Tables S2 and S3). Second, GO analysis reveals that McF LADs, similarly to cLADs, are enriched in genes pertaining to signaling and metabolic functions (Figure S5d), in line with gene ontologies found in LADs in other cell types [20]. Signaling functions are also found in MT-specific LADs, but with lower significance. However, MT-specific LADs also contain genes involved in early developmental processes and cell locomotion (Figure S5d). This finding concurs with the view of stronger lineage commitment of myotubes than fibroblasts, even after their myo-conversion.

**Figure 6:**
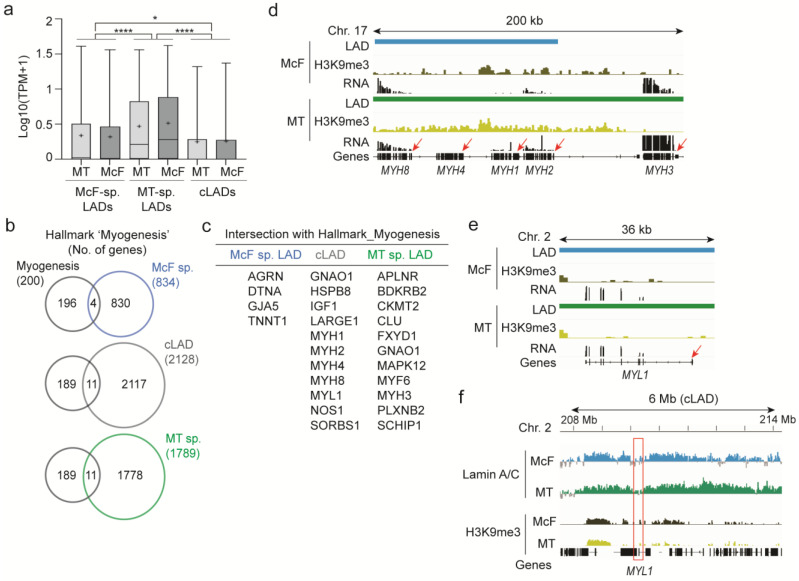
A subset of myogenic genes is expressed in LADs. (**a**) Gene expression levels in McF-specific LADs, MT-specific LADs and cLADs, and for corresponding domains in both cell types; cross, mean; bar, median; box, 25–75% percentile; whiskers, 5–95% percentile; * *p* = 0.023, **** *p* < 0.0001, one-way ANOVA. (**b**) Intersects of genes of the hallmark ‘Myogenesis’ and genes found in McF-specific LADs, MT-specific LADs and in cLADs. (**c**) Genes in intersects shown in (**c**). (**d**,**e**) Genome browser views of LADs, H3K9me3 and gene expression in cLAD sub-domains at the *MHY8-MHY3* cluster (**d**) and *MYL1* gene (**e**). RNA tracks, in counts per million: 0-10 (**d**) and 0-17 (**e**). Arrows point to transcription start sites. (**f**) Genome browser view of lamin A/C and H3K9me3 enrichment in a cLAD containing *MYL1*. Note the lower lamin A/C levels around the gene compared to other LAD regions (red box).

In line with the lower level of H3K9me3 at genes within MT-specific LADs, expression of LAD-associated genes is overall higher, although it remains low, in these LADs than in McF-specific LADs (*p* < 0.0001; one-way ANOVA) or cLADs (*p* = 0.023; one-way ANOVA; Figure 6a). However, most genes making up the hallmark ‘Myogenesis’ discussed above are localized outside LADs in both McF and MT (Figure 6b) and thus reside in a transcriptionally permissive environment; these genes are indeed expressed in the McF and MT systems (see Figure 3c). Nonetheless, a handful reside in cell type-specific LADs or in cLADs (Figure 6c), even though they are expressed in the McF and/or MT systems (see Figure 3c). Thus, their expression cannot be explained by differential lamin A/C association (see, however, below).

As an example, the *MYH8*, *MYH4*, *MYH1*, and *MYH2* clusters are in a cLAD and genes within (*MYH8*, *MYH1*, and *MYH2*) are expressed in either or both MT and McF (Figure 6d; see also Figure 4). None of these genes are enriched in H3K9me3 (Figure 6d), which, as we [25] and others [59] have shown, is compatible with gene expression in LADs. Similarly, the cLAD gene *MYL1* is upregulated upon differentiation in the McF and MT systems (see Figure S2, Cluster 2), which is again likely enabled by local H3K9me3 depletion (Figure 6e,f). The *MYL1* locus is also marked by lower lamin A/C levels than the rest of the LAD (Figure 6f). The localization of myogenic genes in H3K9me3-poor or depleted regions in LADs contrasts with the heterochromatic H3K9me3-rich composition of LADs harboring inactive genes (see Figure 5g,h). These expressed LAD genes are reminiscent of differentially expressed LAD genes identified in cLADs during differentiation of adipose stem cells, which are restricted to local lamin-poor and euchromatin subdomains of LADs [25].

We conclude that (i) while LADs overall share features between McF and MT, LADs unique to MT display weaker heterochromatic features than McF-specific LADs or cLADs. (ii) MT LADs uniquely sequester developmental genes. (iii) A number of myogenic genes are expressed in MT-specific LADs, McF-specific LADs, or in cLADs. This extends the growing evidence of gene activity in LAD subdomains accessible to transcription factors [25] and argues for the regulation of a subset of myogenic genes independently of their localization in LADs, in both systems.

## 4. Discussion

The scarcity of patient samples available when studying a rare disease, the degree of invasiveness of muscle biopsies, and ethical and practical issues in obtaining muscle biopsies from patients with a muscular disorder together prompt a need for a practical and reproducible alternative to investigate phenotypic and genomic aspects of muscle development and pathologies. Skin biopsies constitute, in most cases, a more convenient source of patient material.

We used skin fibroblasts in which ectopic MYOD expression elicits a myogenic program conferring muscle cell features that are similar, in some aspects, to those of myoblast-derived myotubes: (i) phenotype with elongated and polynucleated cells expressing the differentiation marker MHC; (ii) gene expression profiles showing that myo-conversion elicits cell cycle exit and triggers myogenic pathways; (iii) high-order chromatin organization at the level of lamin A/C LADs. As discussed below, there are, however, noticeable distinctions between myo-converted fibroblasts and myoblast-derived myotubes. Importantly, we used isogenic myoblasts and fibroblasts, ruling out for the first time to our knowledge, genetic factors as a source of variations between the MT and McF models and enabling a direct comparison of genomic data between cell types across differentiation replicates.

We compared immortalized fibroblasts from a single 16-year-old donor with immortalized myoblasts from the same donor. Obtaining fibroblasts and myoblasts from the same individual remains a challenge, and our work would benefit from an assessment of the myogenic potential of these cell types in a large number of donors. This would allow us to consider donor age, which affects the proliferative potential of fibroblasts and myoblasts [55], as well as the potential geographical location of the donors (see below). Immortalization is a practical option given the relative scarcity of dual-cell-type donors if one is to carry out multiple and reproducible phenotypic, functional, and epigenomic studies across multiple laboratories. Immortalization of fibroblasts is also necessary for *Myod* transduction and selection to prevent replicative senescence. Immortalization also alleviates the interference of senescence on differentiation, and importantly, it has been shown not to significantly alter the transcriptome of myoblasts and myotubes [38].

A fibroblast-based model presents advantages over reported induced pluripotent stem cell (iPSC)-based models [60]. McFs are simpler (fewer steps and factors), quicker (days vs. weeks), cheaper, and arguably more efficient in generating differentiated cells than iPSCs. As such, McF constitutes an alternative worth exploring further to investigate muscle differentiation and muscle cell function.

There are, however, limitations to the McF system. First, skin fibroblasts are subject to ultra-violet light-induced mutations, arguably more so than myoblasts. Different climates could result in sufficient genotype variations in skin fibroblasts (the donor used in our study lives in a temperate European climate). A heavy mutational load may present potential risks for using skin fibroblasts for biomedical purposes, as shown in skin fibroblast-derived iPS cells [61], and negatively affect differentiation and cell function. Second, as discussed below, because it is a trans-differentiation system, the McF model lacks developmental aspects of myogenesis and shows a delay in myogenic differentiation.

McF displayed a less mature myogenic phenotype than MT on day 5, which was not significantly improved at the transcriptional level by extending differentiation to day 7. In fact, we noted that both multinucleated MT and McF detach from the culture starting around day 5 of differentiation. Our ongoing and unpublished work indicates that this is due to the spontaneous contraction of the differentiated myocytes. Since it occurs with both cell types, this detachment is unlikely to be a direct consequence of an altered differentiation program per se in McF but rather the result of cells being in culture. Myogenic differentiation protocols have been adapted to extend myotube culture over longer periods without cell detachment, including an overlay with a thick layer of Matrigel [62]; this was not applied in our study.

Maturation delay was reflected by the lower expression of markers specific to differentiation stages, including genes of the myosin heavy chain (MHC) family. We also noted a misalignment of nuclei and their clustering in the cell center in McF, contrasting with their alignment in MT or myofibers [63]. This could reflect atypical differentiation and be a sign of potential pathological processes arising from incomplete myogenic differentiation and improper nucleo-cytoskeletal connections [64,65,66,67,68,69,70,71] if it persists at later timepoints [63]. However, nuclear clustering is also an expected phenomenon in the early steps of in vitro muscle differentiation [63,72].

Another factor influencing the extent of myogenic maturation in McF is the differential expression of *MYH* genes, including *MYH1* and *MYH7*, between McF and MT. This might also reflect the ‘natural history’ of muscle ‘stem cells’ used in our systems: in vivo for MT and forced, ex vivo, myogenesis for McF.

At the molecular level, the lack of full myogenic commitment in the McF model may involve the absence of *MYF5* expression. Myogenesis entails chromatin remodeling through histone modifications [73]. MYF5 remodels chromatin by increasing DNA accessibility and acetylates histone H4 at its binding sites, enabling the expression of MYF5 target genes [74]. In turn, at the same sites [74], MYOD also elicits further chromatin remodeling and histone acetylation [75,76,77] and recruits RNA Pol II for activation of muscle-specific genes [74]. Unlike during myoblast differentiation, the McF system bypasses MYF5 [78], so a lack of proper epigenetic priming by MYF5 in the McF system could hamper full developmental myogenic commitment. Improvements in the efficiency of myogenic conversion would entail the induction of early chromatin remodeling and histone acetylation events, recapitulating the early steps of satellite cell and myoblast induction [75,76,77].

Since its publication in 1989 [7], the myo-converted fibroblast model still lacks a rigorous characterization of chromatin organization, including LADs. McF have been compared with undifferentiated myoblasts to postulate that, based on transcriptomic and DNA accessibility patterns, myogenic trans-differentiation of fibroblasts is incomplete [79]. Similarly, 3-dimensional chromatin interactions in myo-converted mouse embryonic fibroblasts remain substantially different from those of C2C12 myoblasts [80]. However, these conclusions are debatable because they rely on comparisons of myo-converted fibroblasts to undifferentiated cells and on cell lines or cell types with different genetic backgrounds.

MT-specific LADs show higher gene density, higher gene expression levels (though this remains low), and weaker heterochromatic features manifested by reduced H3K9me3 than McF-specific LADs or shared LADs. These differences may be explained by the distinct lineage origins of skin fibroblasts (ectoderm) and myoblasts (mesoderm), so that even McF could retain fibroblast characteristics of genome organization. It is remarkable that these genomic properties of MT-specific LADs are similar in McF, in which these domains are not LADs. Conversely, the more pronounced heterochromatic H3K9me3 state of McF-specific LADs is conserved in MT, where these domains are not LADs. This suggests that these properties are not defined by cell type or lamin interactions but rather by gene expression, underlying epigenetic states, or DNA sequence [81,82,83]. Regardless, these findings support increasing evidence of chromatin regulation and gene expression control in LADs [25,84,85] that are uncoupled from association with the nuclear lamina [86,87].

It will be relevant in future studies to monitor LAD repositioning in relation to chromatin states during the actual myo-conversion or differentiation process in isogenic systems to pin-point myogenic-specific mechanisms in these models. Approximately one third of LAD coverage in McF or MT is unique to that cell type. Thus, whether LADs are more divergent in undifferentiated fibroblasts and myoblasts and converge by repositioning after myogenic induction, or whether LADs are already ‘genomically equidistant’ in these cell types and minimally change after myogenic induction, remains to be examined. Gene ontologies associated with McF- or MT-specific LADs further argue that MT uniquely sequesters sets of early developmental and specification genes in LADs, presumably reinforcing the repression of these genes in these terminally differentiated cells [20,25]. The localization of developmental genes in a non-LAD chromatin context in McF may still reflect the potential of fibroblasts to acquire characteristics of other cell types through factor-induced ‘trans-differentiation’ [5,8,88,89,90,91,92].

## 5. Conclusions

Our data globally favor a view of myogenic conversion of human skin fibroblasts as an alternative system to myoblast differentiation and allow us to investigate some aspects of chromatin organization and genome regulation in muscle cells. Our results also point to differences in phenotype, higher-order genome organization, and gene expression between myo-converted fibroblasts and myotubes, the latter not being directly linked to LAD differences.

## Data Availability

Lamin A/C and H3K9me3 ChIP-seq data, and RNA-seq data generated for this study are available at NCBI GEO GSE236120.

## Supplementary Materials

The following supporting information can be downloaded at: https://www.mdpi.com/article/10.3390/cells12151995/s1. Figure S1: GSEA enrichment plot for the Hallmark Epithelial-mesenchymal transition. Figure S2: Genes of the Molecular Signature Database Hallmark Myogenesis, grouped by expression cluster; Figure S3: Prolonged *Myod* induction in fibroblasts does not significantly extend the myogenic gene expression program; Figure S4: LADs shared between McF and MT are also LADs in unrelated fibroblasts; Figure S5: Ontology of genes in McF and MT LADs; Table S1: Pearson correlations of LAD overlap, between differentiation replicates; Table S2: Descriptive statistics of LADs in McF and MT; Table S3: Genes located in McF-only LADs, cLADs and MT-only LADs [30].

## Abbreviations

ChIP	chromatin immunoprecipitation
GEO	Gene expression omnibus
LAD	lamina-associated domain
cLAD	conserved LAD
FB	fibroblast; MB
iPS	Induced pluripotent stem
McF	myo-converted fibroblast
MT	myotube
MHC	myosin heavy chain
PCA	principal component analysis
TPM	transcript per million.
MB	myoblast
seq	sequencing
