# Supplementary material for: Cellular and Genomic Features of Muscle Differentiation from Isogenic Fibroblasts and Myoblasts
Source: Cells. 2023 Aug 3;12(15):1995. doi: 10.3390/cells12151995 (PMC10417614; doi:10.3390/cells12151995)
Supplement: Supplementary file 1 [file cells-12-01995-s001.zip › Benarroch 2023 Supp REVISED_2 230803.pdf]

Supplementary data

Table S1. Pearson correlations of LAD overlap between differentiation replicates.

	LADs (EDD calls) Pearson	
**Replicate**	**McF**	**MT**
1 vs. 2	0.61	0.87
1 vs. 3	0.54	0.88
2 vs. 3	0.78	0.88

Table S2. Descriptive statistics of LADs in McF and MT.

Feature	No. domains	Mean length (Mb)	Coverage (Mb)	No. genes*	Genes/Mb
LADs McF Rep1	696	1.15	798	1995	2.50
LADs McF Rep2	380	1.81	686	1824	2.66
LADs McF Rep3	354	1.72	609	1791	2.94
LADs MT Rep1	675	1.12	753	3278	4.35
LADs MT Rep2	649	1.22	790	3346	4.23
LADs MT Rep3	698	1.05	735	3084	4.19
LADs McF Merged	631	1.45	912	2791	3.06
LADs MT Merged	739	1.18	868	3685	4.24
cLADs	652	0.93	605	2128	3.52
MT-specific LADs	687	0.38	263	1791	6.79
McF-specific LADs	678	0.45	307	834	2.71

*Protein-coding genes.

Table S3. Genes located in McF-only LADs, cLADs and MT-only LADs (separate file).

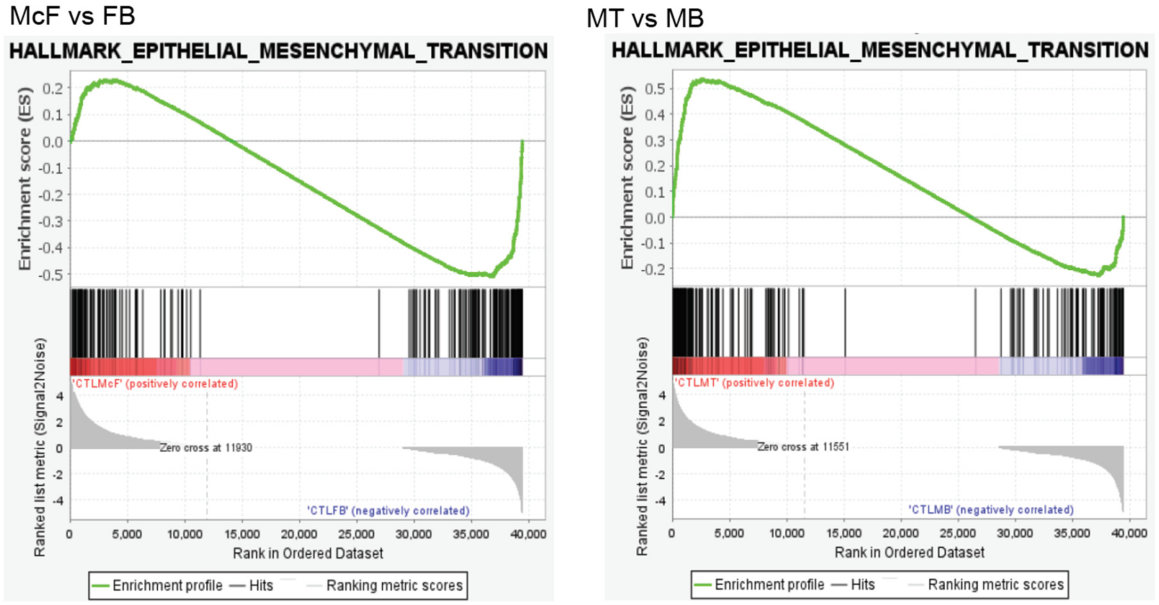

Figure S1. GSEA enrichment plot for the Hallmark “Epithelial-mesenchymal transition, for the McF *vs* FB (left) and MT *vs* MB (right) comparisons. Plots show profiles of the Running enrichment score and positions of gene set members on the rank ordered list (x axis).

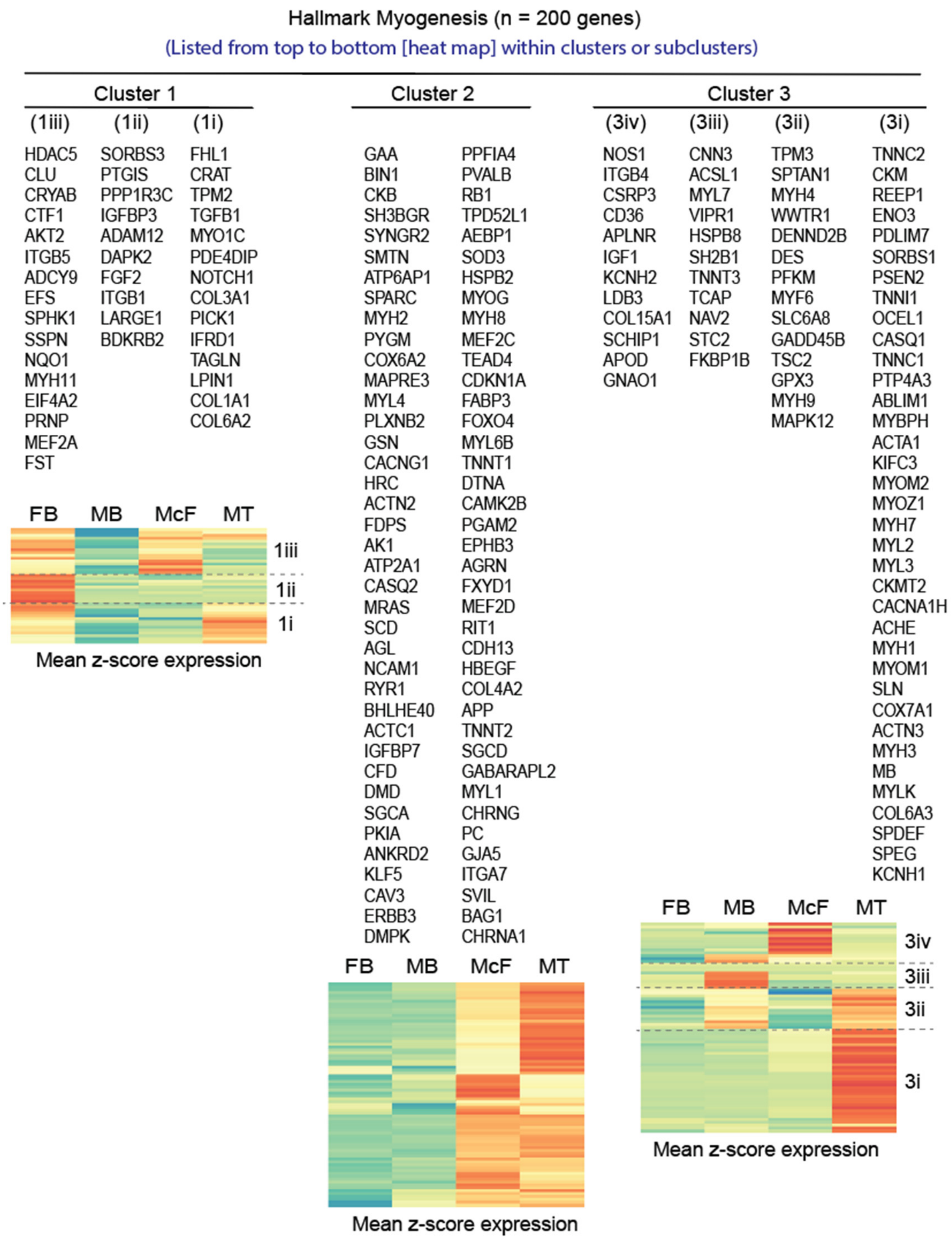

Figure S2. Genes of the Molecular Signature Database Hallmark 'Myogenesis' grouped by expression cluster. Heat maps show mean z-score expression per cluster across the four cell types examined in this study. See Figure 3 for the scale of mean z-score expression.

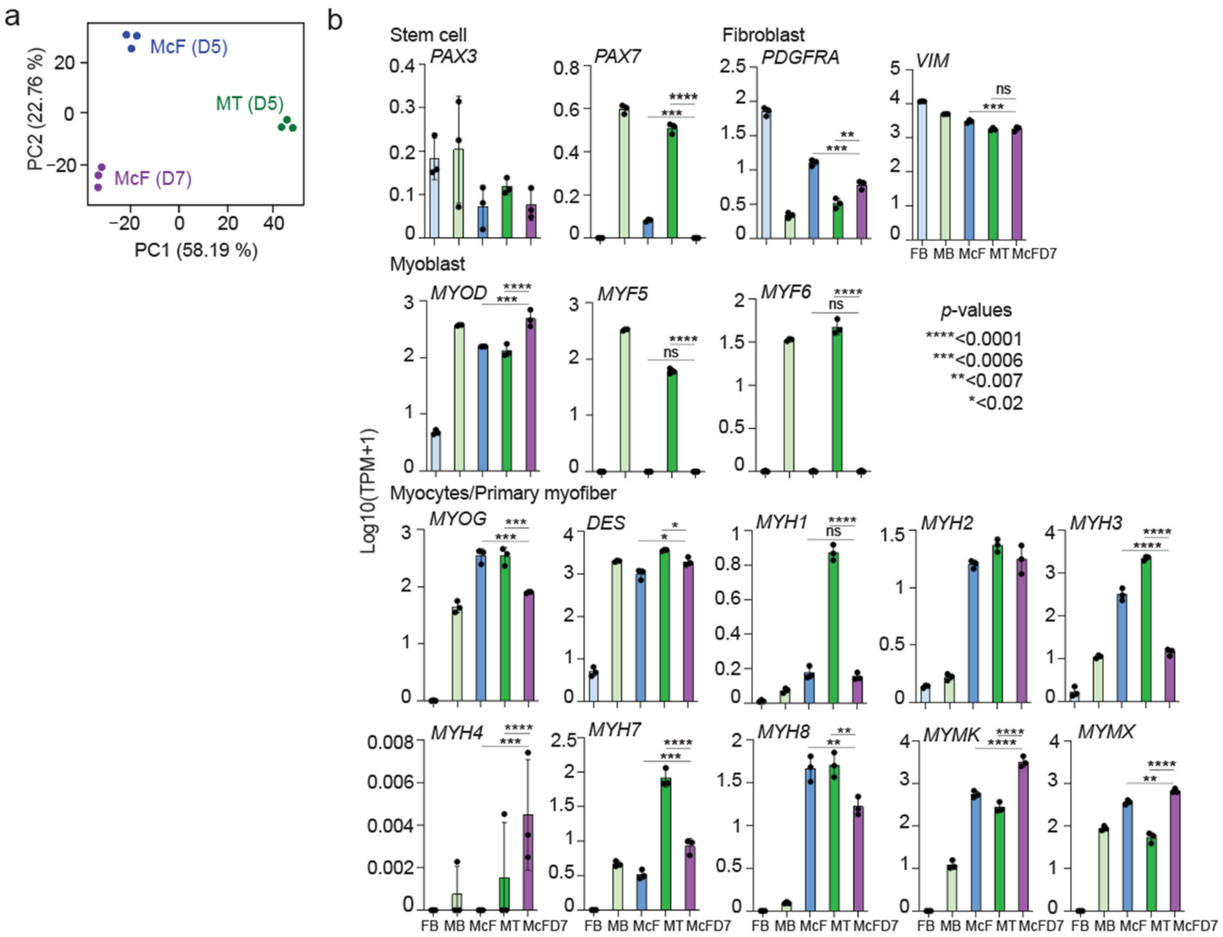

Figure S3. Prolonged *Myod* induction in fibroblasts to 7 days does not significantly extend the myogenic gene expression program. (**a**) PCA plot of gene expression in day-5 McF (McF), day-7 McF (McF D7) and in MT (day 5 as in Figure 2a). (**b**) Expression levels of genes shown in Figure 4, here including day-7 McF (purple bar), from our RNA-seq data for each differentiation replicate (dots); mean ± SD (n = 3). *p*-values: one-way ANOVA.

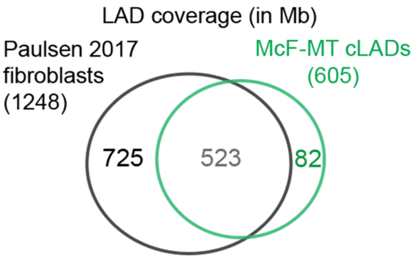

Figure S4. LADs shared between McF and MT are also LADs in unrelated fibroblasts. Venn diagram of overlap (in Mb) between McF-MT cLADs and LADs identified in skin fibroblasts from three unrelated individuals in a previous study (‘Paulsen 2017 fibroblasts’) (ref. [30] in the main text); these LADs consist here of the union of LADs mapped in fibroblasts from these three individuals.

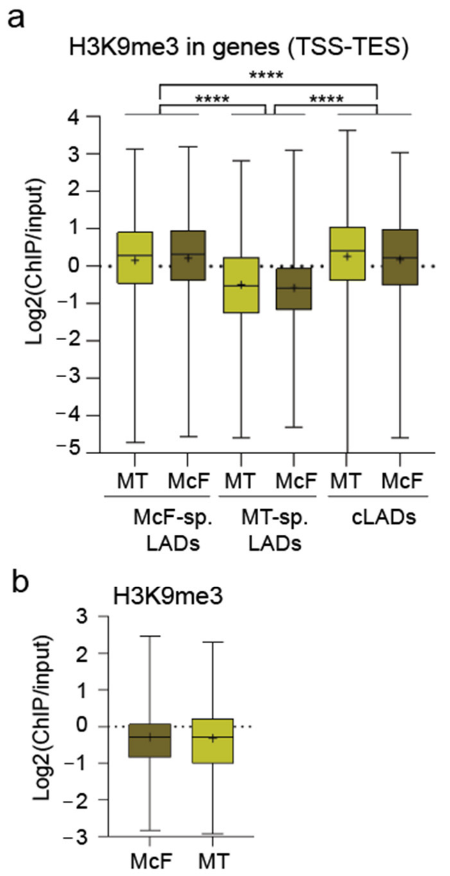

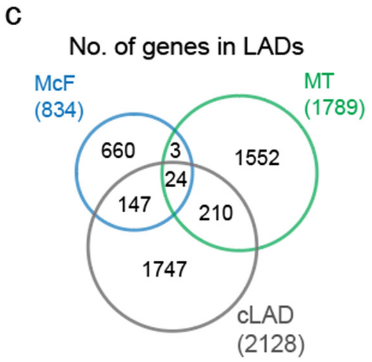

d

GO terms	FDR McF sp. LAD	FDR cLAD	FDR MT sp. LAD
Detection of chemical stimulus	9.80E-20	5.68E-75	1.35E-03
GPCR signalling	1.48E-10	5.54E-63	-
Signal transduction	6.06E-05	1.21E-21	-
Metabolic process	5.18E-05	2.74E-08	-
RNA metabolic process	7.18E-05	2.45E-05	-
Organelle organization	-	1.14E-04	-
Protein metabolic process	-	4.74E-04	-
Biological process	-	-	5.16E-07
Cellular process	4.10E-03	4.41E-04	5.84E-07
Multicellular organism development	-	-	1.53E-04
Embryonic morphogenesis	-	-	1.10E-03
Nervous system development	-	-	1.46E-03
Skeletal system development	-	-	3.16E-03
Locomotion	-	-	5.43E-03

Figure S5. Heterochromatic state and ontology of genes in McF and MT LADs. (**a**) H3K9me3 enrichment at genes (transcription start site [TSS] to transcription end site [TES)) in McF-specific LADs, MT-specific LADs and cLADs, and at the same genes in the other cell type; cross, mean; bar, median; box, 25-75% percentile; whiskers, min-max; ****$p < 0.0001$, ANOVA. (**b**) H3K9me3 enrichment genome-wide in McF and MT. Levels were computed as Log2(H3K9me3 ChIP/input) ratios in 100-kb bins throughout the McF and MT genomes; cross, mean; bar, median; box, 25-75% percentile; whiskers, min-max. (**c**) Venn diagram of overlap of the numbers of genes in McF LADs, MT LADs and in cLADs. (**d**) Gene ontology (GO) terms (PANTHER biological process) associated with all protein-coding genes found in LADs.
