# Supplementary material for: Cellular and Genomic Features of Muscle Differentiation from Isogenic Fibroblasts and Myoblasts
Source: Cells. 2023 Aug 3;12(15):1995. doi: 10.3390/cells12151995 (PMC10417614; doi:10.3390/cells12151995)
Supplement: Supplementary file 1 [file cells-12-01995-s001.zip › Benarroch Table S3.pdf]

Supplementary data

Table S3. Genes located in McF-only LADs, cLADs and MT-only LADs.

McF-only LADs	cLADs	MT-only LADs
SAMD11	TTC34	NPHP4
ABCB11	ZNF214	TMBIM4
AC007846.2	OTUD1	AP000781.2
AC010327.2	OR51G2	SLC2A14
AC020922.1	HBD	CLEC4E
AC087498.1	C10orf113	TNKS1BP1
AC092073.1	C10orf82	TMPRSS13
AC098588.2	OR10G6	FAM71D
AC243967.1	EBF3	KCNJ1
ACAP3	LRRC38	C8A
ACO1	GPC5	AC005697.1
ACP3	OR4D10	EDNRB
ACP6	JAK1	RBMXL1
ACTR3B	GNPTAB	ZFHX3
ACTR3C	ANO4	AC010547.4
ADAM28	PAH	C16orf46
ADAM29	OR8B4	DICER1
ADAM32	LHX5	HSD17B2
ADAMTS20	OR6K3	COA6
ADAMTS3	CARD16	SLC25A21
ADAMTS9	OR8K1	SLC46A3
ADAMTSL3	OR2T1	USH1C
ADGRB3	NCKAP1L	PIGB
ADGRE1	ALDH18A1	CEP126
ADGRF1	TWF1	DUT
ADGRF5	IRAK4	AC090527.3
ADGRL2	KANK4	CTH
ADGRL3	PGR	MBIP
ADGRL4	L1TD1	HHLA3
ADGRV1	GLB1L3	BCL11B
AFDN	TMEM19	XYLT1
AGBL1	OR2T2	OTOG
AGRN	TP73	IGSF21
AKAIN1	MTRNR2L7	MPEG1
AKR1D1	UHRF1BP1L	SLC6A2
AL035425.3	IFI27L1	FADS6
AL357075.4	HELB	VPS33B
AL358113.1	FGF14	ASIC2
AL592490.1	PCNX2	FRMPD2
AL928654.3	OR2AK2	UTF1

ALG14	PGM1	AC093155.3
ALG1L2	GLYAT	PCDH8
AMTN	CASP5	NKX2-8
ANKEF1	CYP2R1	C12orf29
ANKFN1	MASTL	FAM111B
ANKRD30B	CTNNA3	CCDC90B
ANKRD65	CYP4A22	LEPROT
ANKRD66	ADAMTS20	SLC30A4
ANO3	LCE2C	SCYL3
ANP32D	OR6N2	CHRM3
AOC1	SYCP3	PMFBP1
APCS	LRRIQ3	AC093423.3
ARAP2	MS4A6E	FGF14
ARHGAP28	BMS1	PATL1
ARL6IP6	OR2AG1	ETNK1
ARMC4	SETSIP	AL590560.2
ARMH4	GCSAML	HABP2
ARPP21	OR5T1	PAN3
ASB7	ITIH5	MRGPRX1
ASCL4	TNN	ARHGAP21
ASPHD2	OR5AS1	GTF3A
ASTN2	CMA1	TAF15
ATG4A	CCDC197	TMEM104
ATP6V0D2	FZD10	SCO1
ATP6V0E2	GAS2L3	ZNF23
ATP8A1	FAM181B	BIVM
ATP8A2	PROX1	NCOA4
ATRNL1	AMY2A	TMCO1
AURKAIP1	CYP4A11	CACHD1
AVEN	OR2M4	RIC3
AXDND1	MSH4	NTNG1
B3GALT1	OR10A2	TMEM117
B3GALT6	AJAP1	GJB4
BCL9	RAVER2	KYAT3
BEND2	CCDC198	SUPT4H1
BEND4	MS4A12	POGLUT2
BFSP2	OR4D9	GPC6
BNC1	OR2T4	KCNJ11
BRMS1L	RGS7	LRIT2
BRSK1	OR52A5	ZNF705A
BTC	CARD17	MIPOL1
BTLA	OR9G4	CNMD
C12orf42	SELE	KIAA1217
C12orf54	MNDA	FMN2
C14orf177	TRIM58	INPP5A
C14orf28	OPN3	GRID1
C14orf39	OR2G3	SLC18A2

C19orf85	OR51B6	CLEC2D
C1orf159	SMIM1	CSMD2
C1QTNF12	PRAMEF19	GJB3
C2orf83	CYB5R2	MYF6
C4BPA	GBP4	CHIA
C4orf19	MS4A5	CCDC168
C7orf31	SYT1	ERI2
CADM2	OR5M11	TRPC4
CARMIL1	ALG10B	SORD
CASP14	OPALIN	GUCY1A2
CAVIN4	OR4E2	PEX12
CCDC106	TRIM5	OLR1
CCDC140	SYT9	PPP1R12A
CCDC175	OR2C3	NRAP
CCDC60	LAMB3	ZFAND4
CCNA1	SMYD2	MSMB
CCNG1	AC022335.1	ARHGAP11B
CCNL2	CYP4X1	JAK1
CCSER1	CARD18	TTC6
CD200	OR9G1	NHLRC3
CD84	LRRC53	CDC7
CDC14C	PPFIA2	DCUN1D3
CDC42EP5	OR51E1	KCNA1
CDH12	PANX3	TCL1B
CDH19	CYP2C19	IZUMO1R
CDH4	OR4C13	ADGRD1
CDH6	TMEM45B	TUNAR
CDH7	AL583836.1	PANX1
CDH8	C1QL3	ELP4
CDK5RAP2	GZMH	GAS2L2
CEACAM21	CLRN3	PANX3
CEACAM3	GLRX3	C11orf45
CEACAM4	PTPRE	TBRG1
CEACAM5	MGMT	FLI1
CEACAM6	C10orf143	KCNJ5
CEACAM7	MKI67	SIAE
CEP170	ZNF326	SPAG17
CERS6	OR10A4	SLC16A7
CFAP54	SELL	ARMC3
CFAP58	AMY2B	ALDH9A1
CFAP73	PAPPA2	THNSL1
CHD2	PRKCQ	IGSF22
CHRNA7	OR2M5	NLRP10
CLCA1	ITGB3BP	C1orf52
CLCA2	ALG6	SYDE2
CLCA4	EFCAB7	BCL10
CLDN18	CBLIF	GPR180

CLSTN2	OOSP3	ABCC4
CLVS1	WSB2	CFAP52
CNR1	LACRT	HCN4
CNTN5	CD1A	KCTD3
CNTNAP2	SLC17A8	CALB2
CNTNAP3	HS6ST3	LYRM9
CNTNAP5	DCHS1	CMAS
COBL	MGAT4C	C16orf78
COL24A1	ATG4C	ST6GALNAC5
COL4A5	DDX24	FDXR
COL4A6	OTUB2	GRIN2C
COL6A5	OR5AN1	DIAPH3
COL6A6	OR5A2	TDRD3
COX6B2	HBB	CLEC4D
COX7B2	JRKL	TNFSF13B
COX8C	OR11L1	DOCK1
CPNE4	OR4D6	SLAIN1
CPTP	C1orf94	OMA1
CRIM1	CCKBR	MGST1
CRIP1	OR2L8	KNDC1
CRIP2	OR2AJ1	ADGRA1
CRP	FPGT	LRRC8D
CRYBB2	TRIM51	PABPC3
CRYBB3	OR5D16	CENPJ
CSMD1	PMCH	AC092718.8
CSMD3	MTUS2	CDRT15
CXXC4	TMEM225	AP5M1
CYB5A	PDLIM1	TRPC6
CYLD	PTER	IMMP1L
CYP2B6	FOXI2	OR6M1
CYP4F11	SORCS1	NPAT
CYP4F3	SORCS3	ACAT1
CYP4F8	HPSE2	ALKBH8
DACH1	KCNK1	WDFY4
DAOA	SLC35F3	LRRC18
DCAF4L2	ATP8A2	COX10
DCAF8L1	GALC	ABCA9
DCAF8L2	RGS6	FAM20A
DCC	CYP2C18	HEPHL1
DCHS2	OR10G7	DIO2
DDC	RASSF9	PLK1
DDX54	ASTN1	ZNF248
DEFB115	PLEKHA7	RPH3A
DEFB116	PRMT3	OAS1
DEFB118	SLC6A5	OAS3
DEFB119	NELL1	TBX3
DEFB121	ANO5	FBXW8

DEFB123	SLC17A6	TESC
DEPDC1B	NCAPD3	ATG2B
DLG2	FCRL3	C1orf53
DLGAP1	ANKRD30A	DTX4
DMRTA1	SLITRK6	LGALS9
DMRTC2	OR52B4	ARHGAP32
DNER	OR10A6	SLC6A15
DNAAF3	OR51G1	SLC2A3
DOCK3	OR8K3	POMP
DPP6	DRAM1	CDYL2
DTHD1	MS4A7	TPP2
DTNA	NRG3	SLC36A4
DTX1	KIAA0040	PRTFDC1
DUSP27	RABGGTB	PRMT6
DVL1	GRIK3	NFIA
DZIP1L	TCN1	SOX21
EDEM1	OR5J2	CDX2
EFCAB3	ACBD5	FAM111A
ELAVL2	SLITRK5	NOS2
ENPP6	OR8B12	SYNE3
ENTHD1	OR8I2	MTIF3
EPB41L3	ZNF33B	OSBP
EPHA5	CASP4	NKX2-1
EPHA6	OR5M1	FREM2
EPHB1	STX3	DCT
EPN1	OR56B1	GABARAPL1
EPPIN	FSHB	TMEM132B
EPPIN-WFDC6	KCNA4	SUDS3
EPS8L1	OR51F2	DPPA3
ERGIC2	OR6Y1	RAB3GAP2
EYA4	OR9K2	EFL1
EYS	DBX2	PIGBOS1
FABP4	GLT1D1	MYH13
FABP9	TMEM132C	GAS7
FAF1	NFIA	LRRC40
FAM163A	MCOLN2	KCNA2
FAM189A2	FAM155A	SLC35G3
FAM214A	OR2T12	FAR1
FAM71E2	HBG1	AICDA
FAM81B	B3GAT1	BEGAIN
FANCM	MAP1LC3C	TECTB
FAR2	OR10R2	IARS2
FBXO15	CYP2C8	MRE11
FERMT1	PSMA1	LRRIQ1
FGGY	TM2D1	SRSF11
FIGNL1	ALX1	DCTN5
FIZ1	UBQLNL	CLEC12A

FKBP1C	TMEM117	RSL24D1
FKBP3	BECN2	GPAM
FLRT2	OR1C1	GPR26
FMN1	OR2M2	EIF3F
FOLH1	IVL	COP1
FOXA2	C11orf58	CMKLR1
FREM3	OR10S1	GPHN
FRG2	OR8A1	GLRX5
FRG2C	AP002512.3	ALG5
FRK	TESPA1	STARD5
FRMD4B	OR8U1	RFXAP
FSCB	KMO	LRIT1
FSTL5	OR10D3	TSHR
FXN	NALCN	SPACA3
FAAP20	TAL1	PDE4B
G6PC2	ZNF215	AC078927.1
GABRA1	SLCO1B3	GOLGA8S
GABRA2	DEUP1	ABHD13
GABRA4	CNTN5	OR4N2
GABRA6	SLCO1C1	CYFIP1
GABRB1	ARHGAP42	FOXG1
GABRB2	PDE3A	NIPA2
GABRG1	FAT3	LIG4
GABRG2	SLCO1B3-SLCO1B7	GOLGA6L7
GALNTL6	OR8B2	PTPN21
GAPT	SPATA19	BDKRB2
GC	CASP1	PAX9
GCNT1	MYO16	SLFN11
GDAP1	SRRM4	DHRS7C
GIMAP1	UTP20	DHODH
GIMAP1-GIMAP5	ARL1	TXNL4B
GIMAP2	SLC5A8	PKD1L3
GIMAP5	SPIC	HP
GJA5	AK4	GBP3
GJA8	DNAJC6	GBP1
GLRA3	OR8B3	ZC3H14
GLUL	WDR63	DRAM2
GNA14	OR4K2	SLFN12
GNAQ	OR4K5	SLFN13
GNPDA2	CHORDC1	FAM155A
GP6	OR51F1	APOBEC1
GPA33	ACADM	SNX7
GPI	PNLIPRP1	FXYD6
GPM6A	OR8B8	CLMN
GRAP2	OR8H2	LNX2
GRB10	NTS	C16orf82
GRID1	PLPPR5	ITLN2

GRID2	GRIA4	SSTR1
GRIK2	MUCL1	IL16
GRK3	OR5I1	MTMR6
GRM1	TAFA2	SAXO2
GRM5	FCRL1	LAMB3
GRM7	OR5T2	URAD
GRM8	FAM71C	CAPNS2
GUF1	NAALAD2	ARGLU1
H1-7	OR6N1	GPR137B
H2AC1	KIF21A	DUOXA2
H2BC1	ABCD2	DUOXA1
HCN1	OPCML	C14orf132
HELLS	COL11A1	PBX1
HERC2	OR2L13	OR10A3
HES4	TPRG1L	HNRNPCL1
HES5	SPATA6	C1orf141
HMMR	SLCO1A2	NANOGP8
HOOK1	PATJ	ANKRD13C
HPSE2	RNPC3	MGST3
HS3ST1	MS4A2	RAP2A
HSPBP1	OR52E2	AC092111.3
HTR4	RERGL	NIPA1
HTR7	PLPPR4	RGS5
ICA1	TPH2	IQCK
IFTAP	LCE2A	METTL11B
IGF2R	TSPAN8	ST8SIA2
IGSF1	IFI27L2	OTOP2
IKZF1	LRRIQ1	COG7
IL11	AC104389.6	MFAP5
IL1RAPL1	GPR65	ABCA6
IL1RAPL2	KCNK10	ABCA10
ILDR2	LHX8	PTBP2
IMMP2L	APAF1	MMP2
IMPG1	PPP1R1A	NEO1
INTS11	PRDM2	DAB1
IPCEF1	IRAK3	AC068831.8
IQCD	CACNA1E	BMS1
IQGAP2	THYN1	AK7
IRS4	IFI27	USH1G
ISG15	WRAP73	PRAMEF2
ISOC2	OR51B2	KLRB1
JPH1	SUDS3	USP43
JRKL	CD1D	KCNK2
KAZN	CYP2J2	DEPDC1
KCNC2	SELP	SPAG6
KCNIP4	MS4A4A	NALCN
KCNMB4	POGK	COMMD3

KCNU1	OAS1	SDR42E1
KCTD1	LRRTM3	CTSC
KCTD16	GRIN2B	KIF26A
KCTD8	DLG2	ERCC5
KHDRBS3	POU4F1	KSR1
KIF16B	CALCA	TMTC3
KIF25	RAB21	TMC5
KIRREL3	CD1E	ZC3H11B
KIAA0355	PNLIP	FXYD6-FXYD2
KIAA1671	OR5L2	RNF17
KLHL1	MAP3K21	MAPK8
KLHL17	PRDM16	PRAMEF12
KLHL28	CHML	TLL2
KLK4	TRIM21	TMEM45B
KMT5C	OR51V1	CLEC6A
KRBA1	NR1H4	TLE7
KY	MRPL16	TGDS
LAIR1	OR52I2	OPCML
LAIR2	OR51E2	KCNA5
LALBA	AIM2	OR2T10
LAMA1	OR13A1	OR10V1
LENG8	TRIM68	GALNT8
LENG9	OR51D1	KCNA6
LGALS13	TEX36	ZNF202
LGALS16	NPS	OR6X1
LGSN	NELL2	RAB27A
LHFPL3	IKBIP	IRX6
LILRA4	OR52M1	NTM
LILRA5	OR52K1	PRDM10
LILRB2	OR52K2	NFRKB
LINS1	SFMBT2	PTPN5
LMBRD1	PDE1B	CCPG1
LPAR3	DOCK7	ZRANB2
LRATD2	OLFM4	HS3ST3B1
LRFN5	FH	RGR
LRP5L	OR5W2	AMER2
LRRC31	OOSP4B	DNAJC3
LRRC3B	OR5T3	FLT3
LRRC4C	LCE4A	GORAB
LRRC9	OR2G2	PLEKHS1
LRRIQ4	OOSP4A	DZIP1
LRRN3	ANKS1B	LPCAT2
LRRTM4	TRIM3	PYROXD1
LSM14A	PNLIPRP3	FXYD2
LYPD4	TCERG1L	TP53AIP1
MACROD2	CALCB	CEP290
MAEL	SLC44A5	DPYD

MAGEB10	NRXN3	ABCA8
MAGEB18	SLC35F4	AC004687.2
MAGEB5	GPHB5	TBX2
MAGEB6	RHOJ	BCAS3
MAGEB6B	KCNH5	RNF43
MAP2K3	GAD2	ZFP91-CNTF
MAP3K4	AC073612.1	ATF7IP2
MAP3K7	DCD	CFAP161
MARCHF1	OR8G1	GTF2A1
MAST4	VPS26B	GSKIP
MAT2B	IAPP	DPH6
MBD3L2B	TCTN3	MMP13
MBTD1	APBB1IP	CNTF
MCOLN2	ANGPTL3	NEGR1
MCTP1	GLB1L2	WDR25
MDGA2	ZNF496	ZDHHC6
MEP1A	PUS7L	BLOC1S6
MFSD9	AC084337.2	GOLT1B
MITF	OR5R1	SMAD9
MMEL1	ELAVL4	SERBP1
MORN1	CMPK1	TCTEX1D1
MRPL20	CYP4Z1	AK4
MS4A13	LORICRIN	CFHR4
MS4A18	PGLYRP4	CFHR5
MS4A8	PGLYRP3	CFHR2
MSANTD3-TMEFF1	OR4K1	SLFN12L
MTA1	OR2T8	CFAP46
MTR	GBP6	KCND3
MTRNR2L12	OR5M10	UFM1
MTRNR2L13	OR8D4	NAA30
MTRNR2L5	C1orf146	CFAP45
MUC15	LCE2B	KIFAP3
MXRA8	CYP4B1	ROR1
MYO16	COA6	C10orf71
MYO18B	OR10AG1	NUP58
MYO3B	NLRP14	SYT1
NAT14	OR51Q1	CD69
NATD1	MYO3A	ZFP91
NCKAP1L	CADM3	OR2T11
NETO1	CYP2C9	GPR83
NLRP11	OR52N2	KLRC4
NLRP2	MMP26	ANO2
NLRP4	OR52E6	AC068775.1
NLRP7	OR51C1P	NTF3
NLRP9	OR52N4	KLRD1
NOC2L	ACTRT2	C1orf158
NOL4	SH2D4B	MTNR1B

NOS1AP	ERICH3	TGFBR3
NOSTRIN	OR2D2	RXYLT1
NOX3	GRIP1	SLCO3A1
NOX4	KIRREL1	PTPN14
NPHS2	ASB17	VAV3
NPY	ATXN7L3B	ACSM3
NPY2R	VWA5A	CEP128
NRG1	WSCD2	BCO1
NRK	UNC79	MAP2K6
NRXN1	HPX	PDE3A
NSG2	PKP2	MEIS2
NTRK3	ADARB2	TMEM86A
NUDCD2	SLCO1B1	ARHGAP11B
NWD2	MS4A14	METTL21C
NXPE1	CD1C	USH2A
NXPE4	CD1B	RRP15
NYAP2	PPFIBP2	PTPRQ
OCM2	NEDD1	SNX20
ODF2L	FOXD3	WDR63
ONECUT1	OR2M7	SPON1
OPRM1	DPY19L2	AP3B2
OR10A7	ACKR1	CR589904.2
OR10H4	ATRNL1	ATM
OR10J1	LRRC7	LRRC8B
OR10J5	LRRC40	LRRC8C
OR10Q1	SPRR2B	PDC
OR10W1	SPRR2E	PLA2G4A
OR11G2	ZP4	GHITM
OR11H6	MTRNR2L11	C10orf99
OR1A1	MALRD1	OR5AK2
OR1A2	ARL5B	WT1
OR1B1	FANCM	LHX1
OR1D2	CACNB2	AL035078.4
OR1E1	EBLN1	SSRP1
OR1E2	PTF1A	PRG3
OR1G1	NSUN6	RCN1
OR1I1	TLL2	CWF19L2
OR1J2	GZMB	MMP28
OR1J4	STXBP6	C17orf50
OR1L1	MIS18BP1	AATF
OR1L3	TMEM260	LPO
OR1L4	OTX2	MPO
OR1L6	NAA30	TSPOAP1
OR1L8	FOXA1	CCL3L1
OR1N1	NOVA1	HEATR9
OR1N2	MIPOL1	TBC1D3B
OR1Q1	TTC6	CCL4L2

OR1S1	SPRR2A	ODR4
OR1S2	SPRR2D	TPR
OR2AP1	RXRG	SKIDA1
OR2T2	BARHL2	LCE3A
OR2T3	HFM1	DUSP23
OR3A1	NEBL	APLNR
OR3A2	PLXDC2	LRRC55
OR3A3	MSRB2	P2RX3
OR4A47	LCE1A	GPR52
OR4B1	KPRP	MROH9
OR4C3	LCE1C	FMO4
OR4C5	LCE1B	RABGAP1L
OR4K13	EDARADD	SLC18A3
OR4K14	ERO1B	CHAT
OR4K15	GPR137B	DRGX
OR4K17	MT1HL1	OGDHL
OR4L1	MTR	C10orf53
OR4N5	RYR2	MBL2
OR4S1	LCE1D	FMO1
OR4X1	LCE1E	FMO2
OR4X2	LCE1F	FMO3
OR5B12	PRR9	CFHR1
OR5B17	SPRR2F	KCNT2
OR5B2	LELP1	CFHR3
OR5B3	SPRR2G	CFH
OR5BS1P	IFI16	RGS7
OR5D14	SPRR5	RNF2
OR6C1	SLAMF1	SH3BP5L
OR6C2	ITLN1	FAM171A1
OR6C3	CD48	ZNF672
OR6C4	LMX1A	SLC39A12
OR6C6	CD84	LYPD8
OR6C65	LY9	PGBD2
OR6C68	PBX1	MINDY3
OR6C70	NUF2	ITGA8
OR6C74	FCER1A	OR14I1
OR6C75	SLAMF7	ZNF692
OR6C76	CD244	PRKCQ
OR6Q1	SPRR1A	SWT1
OR7D2	CCNJ	DDI1
OR7D4	ZNF518A	MSANTD4
OR7E24	BLNK	KBTBD3
OR7G1	ENTPD1	DCUN1D5
OR7G3	AL365273.2	DYNC2H1
OR8S1	PYHIN1	GREM2
OR9I1	SPRR3	HMCN1
OR9Q1	SPRR4	TRMT1L

OR9Q2	SPRR1B	PRG4
OTOR	SOX6	WSCD2
OVCH1	OR6P1	C1orf115
PAK5	PDE3B	C12orf50
PANK4	SLC5A9	SLC35D1
PAX3	RBMXL2	PAWR
PAXIP1	WASHC3	AC009070.1
PCDH10	OR6T1	CCDC196
PCDH15	BTBD8	VSIG8
PCDH17	RAB3GAP2	RBP3
PCDH20	MARK1	ZNF488
PCLO	CFAP54	NKD1
PCMTD1	NOS1	PIK3R5
PCTP	YME1L1	GLYATL1B
PDCD2L	HSPA12A	IL10RA
PDE1C	ACSS3	REXO5
PDE4D	JAM3	BDKRB1
PDE6H	OR10T2	SLC30A10
PDHA2	NXPE2	SEC23A
PEG3	C11orf42	C12orf60
PERM1	MEGF6	PRAMEF11
PEX10	FOXD2	WDR78
PEX5L	MS4A3	OXGR1
PGGT1B	KLRG1	DLK1
PGM5	OXGR1	TMEM98
PHOX2B	MS4A1	TEX30
PI15	HSPB8	GSG1L2
PID1	OR5P3	ACSS3
PIGT	SVIP	WSB2
PIK3R4	OR5A1	SCEL
PIWIL3	OR4C12	TUBA3C
PKP2	OR10Z1	MTARC1
PLA2G7	PDZRN4	C15orf48
PLBD2	RGS18	FXYD4
PLCH2	TRABD2B	MIER1
PLEKHA7	LCE2D	METTL18
PLEKHB2	MRPL17	ST8SIA1
PLEKHN1	ARHGEF16	PRAMEF1
PLAAT5	FCRL4	DENND1B
PMP2	TMEM132B	RCVRN
POGK	CRYZ	GLMN
POM121L12	C12orf50	ZNF423
POTEC	ZWINT	STX3
POTEE	OR2AG2	C2CD5
PPARGC1A	MS4A4E	ITGBL1
PPDPFL	RBM19	MPHOSPH6
PPHLN1	SPTA1	AL445423.3

PPM1H	FAM78B	DNAJC1
PPP1R12C	OR51S1	CLEC4C
PPP1R21	CAVIN3	LMO3
PPP2R5E	OR6F1	EMX2
PPP4R3C	CCDC196	TBX4
PPP6R1	OR52J3	CLEC4A
PRAMEF14	OMA1	WLS
PRAMEF15	DAB1	DIRAS3
PRAMEF33	FAF1	GNG12
PRDM2	HOOK1	RPE65
PRDM9	BARX2	TCL1A
PRICKLE1	OR6K2	PCNX2
PRKCZ	PDZK1IP1	LEPR
PRPF39	EXO1	ADRA2A
PRR20A	USH2A	ANTXRL
PRR20B	GPATCH2	PTPN20
PRR20C	ESRRG	GPRIN2
PRR20D	SPATA17	GDF10
PRR20E	RRP15	GDF2
PRXL2B	BEND5	IL12RB2
PSKH2	PIWIL1	ADPRM
PSMD10	ASB2	NAT9
PSMD7	SLC39A12	PAX6
PTPRB	ATP1B1	COMMD3-BMI1
PTPRD	OBI1	AC015688.4
PTPRH	OR51A2	C3AR1
PTPRJ	C1orf68	PRRX1
PTPRO	OR10K1	BPNT1
PTPRR	F5	BMI1
PURG	C12orf42	GCSH
PUSL1	PDPN	C8B
PXDNL	FBXO21	PIK3R6
RAB28	MS4A6A	TMTC4
RAB38	FCRL2	LHX9
RAB3C	IGSF9B	AL355102.2
RALYL	TMEM132D	MYH3
RASAL1	TNR	ENKUR
RASGRF2	ACAD8	PAPOLA
RBFOX1	ITIH2	HTATIP2
RBM33	NUP37	CMC2
RDH13	OR52R1	GDF3
RELN	SLC25A3	IRX5
RER1	FOXE3	INSL5
RERG	OR10K2	EPRS1
RFPL4A	OR52N5	KLRK1
RFPL4AL1	OR52N1	KLRC4-KLRK1
RGPD3	RRP8	AC010197.2

RGPD4	ILK	LDHB
RGS21	GBP7	CHI3L2
RGS6	OR14K1	PRLHR
RGSL1	PKN2	DENND2D
RIT2	GRID1	DEUP1
RITA1	BRINP2	ZNF25
RNF175	OR10G9	C14orf178
RNF223	CCDC27	KLHDC7A
ROBO1	OR5M8	CSNK1A1L
ROBO2	OR5M3	SUPT20H
RORA	OR14C36	PSMA1
RP1	RFC5	STX8
RPL10L	PLD5	ACSL5
RPL28	HBE1	PHC1
RPS6KC1	GBP5	PIFO
RSU1	EPHX4	SLAMF8
RTN1	GCSAML-AS1	CASP7
RWDD3	CACHD1	GTF2B
RYR3	OR2M3	TUB
S100A7A	LEPROT	GBP2
S100A7L2	LEPR	GBP7
SAMD5	AVPR1A	CPEB1
SAMD7	OOSP2	UGGT2
SBK2	OR51I2	CLEC2B
SBK3	OR52D1	KLRF2
SCGB2B2	VAX1	SCN4B
SCGN	CPNE8	DUOX2
SCN11A	OR8H1	FLT1
SCNN1D	PRAMEF20	DLGAP3
SCYGR5	OVCH2	MYF5
SDF4	C1orf174	GJB5
SDK1	TBC1D15	GDE1
SDS	KCNT2	ALOX5
SDSL	CAMK1G	MARCHF8
SERPINA7	PRIMA1	RAB37
SETMAR	OR8K5	PDX1
SEZ6L	OR5F1	GPR12
SFRP2	OR10G8	ADCK1
SGCZ	IGF1	ATMIN
SGPP2	OLFML1	OTOGL
SGSM1	OR5L1	TPTE2
SH2D4B	DPYD	IGSF9
SH3GL3	OR2T6	ABCC8
SHH	PARPBP	CENPN
SHISA3	MS4A13	BIVM-ERCC5
SHISA7	OR51B5	KLRG1
SHISA9	ATP5F1C	FANCF

SKI	STIL	SGIP1
SLAMF6	TNNI3K	ZNF644
SLC16A9	GLMN	TAGLN2
SLC17A1	SLC2A13	SHF
SLC17A3	LRRK2	SLC28A2
SLC17A4	C12orf40	DUOX1
SLC19A3	OR5P2	LIN7A
SLC22A1	LGR5	NR2F2
SLC22A10	S100A12	ASPM
SLC22A2	ZFC3H1	PCSK6
SLC22A24	S100A9	F13B
SLC22A25	S100A8	ZBTB41
SLC22A3	THAP2	GRIN2A
SLC22A9	FCRL5	CRB1
SLC25A27	MUC19	GATM
SLC26A5	TMPO	NOD2
SLC27A6	A2M	DIO3
SLC35F1	NEUROD4	TMC3
SLC35F4	OR2B11	TCF7L2
SLC4A10	OR6A2	GXYLT1
SLC7A13	RIMBP2	TMEM220
SLC8A3	OR14A2	FAM204A
SLC8B1	BRINP3	RASGEF1A
SLC9A2	ARFIP2	RECQL
SLC9C1	OR5AR1	PROSER1
SMAD5	PZP	ASPG
SMIM17	OR56B4	H4-16
SMIM31	OR8G5	STON2
SMIM36	ABI1	GLYATL2
SMOC1	AC118470.1	RAB11FIP2
SNRPB2	INSC	KITLG
SNRPN	OR2L3	TUBGCP2
SNTG1	TBX5	MFSD6L
SOX17	KSR2	NTN1
SPATA22	C10orf67	PRG2
SPATA48	TMTC2	SCNN1B
SPC25	OR2D3	SRGAP1
SPINT3	LUZP2	PEBP1
SPOCK3	OR8D2	SPATA7
SSC5D	OR51I1	KLRF1
SSU72P2	AMY1C	NME7
SSU72P3	KCNA3	CCDC181
SSU72P4	KCNA2	BLZF1
SSU72P5	AMY1B	ATP1B1
SSU72P7	CD53	SLC19A2
STK31	TRHDE	GPRC5B
STK33	LRIF1	C1orf112

STON1	SMPD1	RERGL
STON1-GTF2A1L	APBB1	PIK3C2G
STPG2	CADM1	EXOC5
STXBP6	CHRM3	CDHR1
SULT1C2	TPP1	ABCC9
SULT1C3	TAF10	KCNJ8
SUMF1	OR8J3	GSX1
SVOPL	DEPDC4	HYDIN
SYT1	C1orf112	PIP4K2A
SYT16	OR13G1	PDZD8
SYT4	HELLS	SMCO4
SYT5	OR51A4	FOXJ2
TADA1	TYW3	RPAP2
TAFA2	LRRC52	MLLT10
TAFA4	OR8J1	MTUS2
TAS1R3	SMIM12	C1orf87
TBC1D12	OLFM3	NUF2
TBC1D22A	OR8H3	POLR1D
TDRD5	ST6GALNAC3	KCNA10
TDRD6	CNTN1	SPATA5L1
TECRL	TRPC6	SFTA3
TEDC1	OR2L5	VENTX
TEDDM1	MCOLN3	CEPT1
TENM2	SOX5	C15orf41
TEX13A	SPATA7	ABCA5
TEX15	ASCL1	AC092718.3
TEX22	OR2W3	NKX6-2
TEX36	AMY1A	UCK2
TGFBRAP1	DNHD1	GYS2
TIMM21	ACSM6	ANKRD49
TJP2	SLC10A2	UNC45B
TLCD4	ROR1	LMO4
TLCD4-RWDD3	UBE2U	PKN2
TLL1	OR8D1	SEL1L
TMCO5A	OR2T33	LMO1
TMEFF1	OR10G2	SLFN14
TMEM100	ANKRD26	GLYATL1
TMEM108	OR4D11	GPC5
TMEM11	GPR158	LPXN
TMEM114	KIN	PRMT3
TMEM121	OR2L2	ADAM8
TMEM150B	OR52A1	FAM90A1
TMEM176A	CHPT1	DHX38
TMEM176B	MYBPC1	HPR
TMEM178B	SCYL2	CMTR2
TMEM182	TIMM10B	SPX
TMEM190	HBG2	RIMKLB

TMEM211	OR5D18	ATP12A
TMEM238	AC104389.5	A2ML1
TMEM260	NLRP3	VTI1A
TMEM38B	OR4E1	AP2B1
TMEM86B	OR51L1	NECAP1
TMTC1	OR10X1	MTARC2
TNFRSF14	AGBL4	IL23R
TNFRSF18	CEP104	C1orf94
TNFRSF4	DFFB	SMIM12
TNNI3	OR51A7	NANOG
TNNT1	OR51T1	NANOGNB
TOGARAM1	WDR64	HPSE2
TOP1	GAS2	FBXO21
TPCN1	ZNF648	RET
TRAM1L1	OR4D5	FUT8
TRIM24	ACTR6	GNAO1
TRIM32	CTSG	RASL10B
TRIM42	OOSP1	CLDN10
TRIM49B	AL162596.1	MRPS14
TRIM51GP	LCE6A	CACYBP
TRIM64C	SMCP	TNR
TRIM77	AL138899.3	CENPF
TRIP12	STK33	TSPAN19
TRPM3	TNFSF13B	SLFN5
TSHZ2	IMMP1L	RAN
TTC29	OR10G4	MPP5
TTC34	DMRTA2	GADD45A
TTLL10	LRRC47	HMGB4
TTLL7	USP1	PTGER3
TTYH1	OR52I1	AC005833.1
TYR	CD5L	SMYD2
U2AF2	TRIM22	TMEM52B
UBA2	ENO4	SMIM35
UBE2J2	PRAMEF17	GJA4
UBE2S	OR51B4	M6PR
UNC5C	CWF19L2	CLEC14A
UNC5D	RPH3A	PLCG2
UNC79	OR14A16	CPXM2
USP29	FAM160A2	SMCO3
USP31	GATA3	ANO3
UTP18	PDSS1	GLYAT
VANGL2	FPGT-TNNI3K	ZNF326
VAV1	SORBS1	ANGPTL5
VSIG1	FAM181A	SLC9A3R1
VWC2	CCDC59	LYRM1
WDR37	BRDT	FCRL6
WFDC10A	DCDC1	GLT1D1

WFDC11	DNAJC24	STX2
WFDC2	CCDC179	VSIG10
WFDC6	ANO3	TAOK3
WFDC8	ARL14EP	TMEM132C
WFDC9	MPPED2	SLC15A4
WSCD1	KIAA1217	OR5B21
WTIP	SHTN1	TMPRSS4
WWP1	NUP58	HS3ST3A1
XIRP2	OR10A5	TWF1
XYLT1	TAF3	GAS2
YIPF7	GRM5	EFNB2
ZFP28	OR52L1	STYK1
ZIM2	OR52W1	WBP11
ZIM3	CNGA4	ART4
ZNF181	PLPP4	JAML
ZNF30	FGFR2	SCN3B
ZNF302	KCNK18	SCN2B
ZNF317	CC2D2B	PDGFD
ZNF470	OR56A4	PLBD1
ZNF471	OR56A5	MAGOHB
ZNF524	OR52H1	CLEC1B
ZNF579	UBQLN3	CLEC2A
ZNF580	TRIM6-TRIM34	CLEC1A
ZNF581	TRIM34	CLEC7A
ZNF582	OR52E4	KLRC2
ZNF583	OR52E5	KLRC1
ZNF599	WDR11	GRAMD1B
ZNF628	OR51M1	CLECL1
ZNF641	OR6K6	EDARADD
ZNF667	OR56A3	EIF2S3B
ZNF682	CCDC172	DSCAML1
ZNF699	DNTT	AASDHPPT
ZNF704	CCDC60	GLP2R
ZNF71	OR56A1	GUCY2C
ZNF717	OR5M9	EXOSC8
ZNF784	TRIM6	CLEC9A
ZNF835	OR52B2	H2AJ
ZNF865	OR52B6	CLEC12B
ZNF93	GFRA1	CEP164
ZPBP	METTL25	DNAH3
ZPLD1	OR5AP2	STOML3
ZSCAN5A	OR52E8	KLRC3
	PPP4R4	ENGASE
	SERPINA10	RBFOX3
	SERPINA6	LDLRAD4
	SERPINA1	FAM210A
	SERPINA11	RNMT

SERPINA9	PSMA8
SERPINA12	TAF4B
SERPINA4	KCTD1
SERPINA5	TRAPPC8
AL049839.2	RNF125
SERPINA3	RNF138
GSC	GAREM1
VRK1	MEP1B
GOLGA6L2	KIAA1328
MKRN3	CELF4
MAGEL2	MYO5B
NDN	CFAP53
NPAP1	MBD1
GABRB3	CXXC1
GABRA5	SKA1
GABRG3	MAPK4
OCA2	MBD2
HERC2	POLI
GOLGA6L7	STARD6
APBA2	C18orf54
FAM189A1	BOD1L2
NSMCE3	SERPINB2
FAN1	SERPINB10
MTMR10	HMSD
TRPM1	SERPINB8
KLF13	TMX3
OTUD7A	CCDC102B
CHRNA7	DOK6
TMCO5A	CD226
AC090527.3	RTTN
SQOR	SOCS6
SEMA6D	DIPK1C
SLC24A5	CNDP2
MYEF2	CNDP1
CTXN2	ZNF558
SLC12A1	MBD3L1
ONECUT1	ZNF333
WDR72	ZNF254
UNC13C	VSTM2B
FOXB1	POP4
MEX3B	PLEKHF1
NTRK3	C19orf12
SV2B	CCNE1
CHD2	URI1
RGMA	ZNF536
MCTP2	ZNF507
ARRDC4	DPY19L3

ADAMTS17	PDCD5
CERS3	ANKRD27
LINS1	RGS9BP
RBFOX1	NUDT19
TMEM114	TDRD12
GRIN2A	SLC7A9
SYT17	CEP89
CLEC19A	FAAP24
TMC5	RHPN2
GPR139	GPATCH1
GP2	WDR88
UMOD	LRP3
PDILT	SLC7A10
ACSM5	CEBPA
ACSM2A	CEBPG
ACSM2B	GRAMD1A
ACSM3	SCN1B
ACSM1	HPN
THUMPD1	AC020907.6
ERI2	FXYD3
HS3ST2	LGI4
PLK1	FXYD1
ERN2	FXYD7
CHP2	FXYD5
PRKCB	RPS19
CACNG3	CD79A
HS3ST4	ARHGEF1
CBLN1	ERFL
C16orf78	RABAC1
SALL1	AC010616.1
HNRNPA1P48	ATP1A3
TOX3	GRIK5
SLC6A2	ZNF175
CES1	ZNF83
CES5A	ZNF611
GNAO1	ZNF600
CDH8	ZNF28
ZFHX3	ZNF468
CNTNAP4	ZNF320
AC106741.2	ZNF888
MON1B	ZNF816-ZNF321P
SYCE1L	ZNF816
ADAMTS18	ERVV-1
NUDT7	ERVV-2
VAT1L	ZNF160
CLEC3A	ZNF415
WWOX	ZNF347

MAF	ZNF665
DYNLRB2	ZNF677
CDYL2	VN1R2
MYH8	VN1R4
MYH4	ZNF845
MYH1	ZNF525
MYH2	ZNF765
TMEM238L	AC022137.3
PIRT	ZNF761
SHISA6	ZNF813
DNAH9	ZNF331
ZNF18	LRATD1
MAP2K4	NBAS
MYOCD	DDX1
ARHGAP44	GDF7
ELAC2	LDAH
KCNJ12	CAPN13
KCNJ18	GALNT14
MTRNR2L1	CAPN14
ASIC2	EHD3
CCL2	CCDC85A
CCL7	BCL11A
CCL11	EHBP1
CCL8	OTX1
CCL13	MEIS1
CCL1	ETAA1
TMEM132E	C1D
CCT6B	WDR92
ZNF830	AC017083.3
LIG3	MRPL19
RFFL	GCFC2
AC004223.3	SUCLG1
RAD51D	EIF2AK3
FNDC8	DDX18
NLE1	CCDC93
TAF15	INSIG2
HEATR9	EN1
CCL5	MARCO
RDM1	HS6ST1
LYZL6	CCDC74B
CCL16	SMPD4
CCL14	MZT2B
CCL15-CCL14	TUBA3E
CCL15	CCDC115
CCL23	IMP4
CCL18	PTPN18
CCL3	GPR39

CCL4	LYPD1
UTP18	NCKAP5
CA10	ZRANB3
KIF2B	LCT
TOM1L1	MCM6
COX11	DARS1
STXBP4	ARHGAP15
HLF	KIF5C
MMD	LYPD6B
SMIM36	LYPD6
ANKFN1	NR4A2
NOG	GPD2
SOX9	GALNT5
SLC39A11	DPP4
SSTR2	GCG
COG1	FAP
FAM104A	IFIH1
C17orf80	GCA
CPSF4L	KCNH7
CDC42EP4	FIGN
SDK2	GRB14
RPL38	COBLL1
TTYH2	SLC38A11
DNAI2	EVX2
KIF19	HOXD13
BTBD17	HOXD12
GPR142	HOXD11
GPRC5C	HOXD10
CD300A	HOXD9
CD300LB	HOXD8
CD300C	HOXD3
CD300LD	HOXD4
CD300E	HOXD1
RAB37	MTX2
CD300LF	PPP1R1C
EPB41L3	PDE1A
TMEM200C	DNAJC10
L3MBTL4	NCKAP1
ARHGAP28	DUSP19
MC5R	ZC3H15
MC2R	ITGAV
ZNF519	FAM171B
KCTD1	ZSWIM2
AQP4	TMEFF2
CHST9	SLC39A10
DSC3	DNAH7
DSC2	PLCL1

DSC1	SATB2
DSG1	FTCDNL1
DSG4	DYTN
DSG3	MDH1B
DSG2	FASTKD2
TTR	PIKFYVE
B4GALT6	PTH2R
SLC25A52	IKZF2
TRAPPC8	SPAG16
KLHL14	VWC2L
CCDC178	BARD1
ASXL3	ABCA12
NOL4	ATIC
CELF4	FN1
RIT2	HAO1
HMSD	TMX4
SERPINB8	PLCB1
CBLN2	SPTLC3
MUC16	ISM1
OR1M1	TASP1
OR7G2	SEL1L2
ZNF333	MACROD2
ADGRE2	PCSK2
OR7C1	DZANK1
OR7A5	POLR3F
OR7A10	RBBP9
OR7A17	SEC23B
OR7C2	SMIM26
SLC1A6	AL121900.1
CCDC105	DTD1
CYP4F12	SCP2D1
OR10H2	SLC24A3
OR10H3	RALGAPA2
OR10H5	KIZ
OR10H1	NKX2-2
CYP4F2	CST4
ZNF682	CST1
ZNF90	CST2
ZNF486	CST5
ZNF737	GGTLC1
ZNF626	FAM83D
ZNF66	DHX35
ZNF85	CD40
ZNF430	CDH22
ZNF714	SLC35C2
ZNF431	ZFP64
ZNF708	CYP24A1

ZNF738	PFDN4
ZNF493	DOK5
AC010615.4	CSTF1
ZNF429	CASS4
ZNF100	RTF2
ZNF43	GCNT7
ZNF208	FAM209A
ZNF257	FAM209B
ZNF676	TFAP2C
ZNF729	BMP7
ZNF98	SPO11
ZNF492	ZNF831
ZNF99	CDH4
ZNF723	BACH1
ZNF728	GRIK1
ZNF730	TIAM1
ZNF724	HUNK
AC092329.3	B3GALT5
ZNF91	IGSF5
ZNF675	BACE2
ZNF681	ABCG1
ZNF726	TFF3
ZNF254	Z82190.2
UQCRFS1	YWHAH
VSTM2B	FBXO7
ZNF536	SYN3
TSHZ3	TIMP3
PEPD	HMGXB4
CHST8	SCUBE1
KCTD15	MPPED1
KLK5	EFCAB6
KLK6	WNT7B
KLK7	BRD1
KLK8	PANX2
AC011473.4	TRABD
KLK9	SELENOO
KLK10	TUBGCP6
KLK11	HDAC10
KLK12	MAPK12
KLK13	MAPK11
KLK14	PLXNB2
CTU1	DENND6B
SIGLEC9	IQSEC1
SIGLEC7	NUP210
CD33	HDAC11
SIGLECL1	FBLN2
IGLON5	WNT7A

VSIG10L
ETFB
AC008750.8
CLDND2
NKG7
LIM2
C19orf84
SIGLEC10
SIGLEC8
CEACAM18
SIGLEC12
SIGLEC6
DPRX
LILRA2
LILRA1
LILRB1
LILRB4
KIR3DL3
KIR2DL3
KIR2DL1
KIR2DL4
KIR3DL1
KIR3DL2
FCAR
NCR1
NLRP7
NLRP2
NLRP4
NLRP13
NLRP8
NLRP5
ZNF787
ZNF444
GALP
ZSCAN5B
ZSCAN5C
ZSCAN5A
EDDM13
FAM110C
SH3YL1
ACP1
ALKAL2
TMEM18
SNTG2
TPO
PXDN
MYT1L
TBC1D5
SATB1
KAT2B
SGO1
ZNF385D
RARB
TOP2B
NGLY1
OXSM
CMC1
STAC
DCLK3
TRANK1
SCN11A
WDR48
GORASP1
TTC21A
CSRNP1
XIRP1
CX3CR1
CCR8
SLC25A38
RPSA
MOBP
MYRIP
EIF1B
ENTPD3
RPL14
ZNF619
ZNF620
ZNF621
CACNA1D
CACNA2D3
ERC2
FHIT
CADPS
SYNPR
SNTN
KBTBD8
SUCLG2
CNTN3
ROBO2
ROBO1
GBE1
CHMP2B
POU1F1
ARL13B

EIPR1	STX19
TRAPPC12	DHFR2
ADI1	NSUN3
AC108488.2	CLDND1
RNASEH1	AC021660.3
RPS7	GPR15
COLEC11	CPOX
ALLC	MYH15
DCDC2C	CIP2A
SOX11	DZIP3
CMPK2	LSAMP
RSAD2	IGSF11
RNF144A	NDUFB4
MYCN	HGD
CYRIA	RABL3
LDAH	GTF2E1
APOB	STXBP5L
TDRD15	CSTA
STON1	CCDC58
STON1-GTF2A1L	ATP2C1
GTF2A1L	ASTE1
AC073082.1	NEK11
LHCGR	NUDT16
FSHR	MRPL3
NRXN1	CPNE4
VRK2	PLOD2
FANCL	PLSCR4
BCL11A	PLSCR2
LRRTM4	PLSCR1
REG3G	PLSCR5
REG1B	MED12L
REG1A	P2RY14
REG3A	GPR87
CTNNA2	P2RY13
LRRTM1	P2RY12
FOXI3	IGSF10
TEX37	AADACL2
RPIA	AADAC
TMEM182	SUCNR1
POU3F3	RAP2B
MRPS9	MME
GPR45	STRIT1
TGFBRAP1	PLCH1
CD8B2	C3orf33
ST6GAL2	AC104472.3
SLC5A7	SLC33A1
DPP10	GMPS

RAB6C	KCNAB1
GPR148	VEPH1
AMER3	PTX3
ARHGEF4	SLC66A1L
SMIM39	SHOX2
FAM168B	RSRC1
PLEKHB2	IQCJ-SCHIP1
RAB6D	SCHIP1
MZT2A	IL12A
TUBA3D	C3orf80
CCDC74A	AC079594.2
ANKRD30BL	IFT80
CXCR4	SMC4
THSD7B	TRIM59
HNMT	KPNA4
SPOPL	NCEH1
NXPH2	ECT2
LRP1B	NLGN1
KYNU	NAALADL2
ARHGAP15	CLDN1
RPRM	CLDN16
GALNT13	TMEM207
KCNJ3	IL1RAP
NR4A2	GMNC
GPD2	OSTN
SLC4A10	UTS2B
DPP4	CCDC50
KCNH7	PYDC2
FIGN	ATP13A4
GRB14	OPA1
SCN3A	HMX1
SCN2A	DEFB131A
CSRNP3	SLC2A9
GALNT3	DRD5
TTC21B	WDR1
SCN1A	NKX3-2
SCN9A	BOD1L1
SCN7A	FGFBP1
XIRP2	FGFBP2
DHRS9	PROM1
LRP2	LDB2
DUSP19	QDPR
NUP35	CLRN2
ZNF804A	LAP3
FSIP2	MED28
SLC39A10	FAM184B
DNAH7	DCAF16

PTH2R	NCAPG
MAP2	LCORL
UNC80	SLIT2
RPE	PACRGL
KANSL1L	KCNIP4
ACADL	PCDH7
MYL1	DCUN1D4
LANCL1	KIT
CPS1	EXOC1
ERBB4	CEP135
SPAG16	CRACD
SLC19A3	ADAMTS3
SCYGR6	CFAP299
SCYGR7	BMP3
SCYGR8	PRKG2
CCL20	RASGEF1B
DAW1	GPRIN3
SPHKAP	SNCA
PID1	MMRN1
DNER	UNC5C
BMP2	ADH4
PCSK2	ADH6
SLC24A3	ADH1A
RALGAPA2	ADH7
PAX1	C4orf17
MAFB	TRMT10A
PTPRT	MTTP
TSHZ2	C4orf54
DOK5	DAPP1
CBLN4	LAMTOR3
MC3R	DNAJB14
FAM210B	H2AZ1
AURKA	SLC9B2
CSTF1	BDH2
ZNF831	CENPE
EDN3	MAD2L1
PHACTR3	PRDM5
SYCP2	NDNF
FAM217B	TNIP3
PPP1R3D	QRFPR
CDH26	ANXA5
CDH4	TMEM155
LIPI	EXOSC9
RBM11	CCNA2
HSPA13	BBS7
SAMSN1	TRPC3
CXADR	KIAA1109

BTG3	ADAD1
C21orf91	FAT4
CHODL	INTU
TMPRSS15	SLC25A31
NCAM2	HSPA4L
GRIK1	PLK4
CLDN17	MFSD8
CLDN8	ABHD18
KRTAP24-1	LARP1B
KRTAP25-1	MGAT4D
KRTAP26-1	ELMOD2
KRTAP27-1	UCP1
KRTAP23-1	TBC1D9
KRTAP13-2	RNF150
KRTAP13-1	ZNF330
KRTAP13-3	AC098588.2
KRTAP13-4	HHIP
KRTAP15-1	ANAPC10
KRTAP19-1	LSM6
KRTAP19-2	REELD1
KRTAP19-3	SLC10A7
KRTAP19-4	TTC29
KRTAP19-5	MAP9
KRTAP19-6	APELA
KRTAP19-7	TRIM61
KRTAP22-2	FAM218A
KRTAP6-3	TRIM60
KRTAP6-2	TRIM75P
KRTAP22-1	TMEM192
KRTAP6-1	CPE
KRTAP20-1	FBXO8
KRTAP20-4	CEP44
KRTAP20-2	HPGD
KRTAP20-3	GLRA3
KRTAP21-3	AGA
KRTAP21-2	TRAPPC11
KRTAP21-1	ADAMTS16
KRTAP8-1	ICE1
KRTAP7-1	MED10
KRTAP11-1	UBE2QL1
KRTAP19-8	NSUN2
TIAM1	SRD5A1
PCP4	TENT4A
DSCAM	ADCY2
SLC5A1	C5orf49
C22orf42	MTRR
RFPL2	FASTKD3

SLC5A4	SEMA5A
RFPL3	TAS2R1
RTCB	SPEF2
BPIFC	OXCT1
SYN3	C5orf51
LARGE1	FBXO4
ISX	GHR
TBC1D22A	ERCC8
TAFA5	NDUFAF2
CHL1	KIF2A
CNTN6	IPO11
CNTN4	RGS7BP
IL5RA	SHISAL2B
TRNT1	MAST4
CRBN	CD180
SUMF1	SLC30A5
LRRN1	CCNB1
SETMAR	CENPH
GRM7	MRPS36
KCNH8	CDK7
EFHB	CCDC125
RAB5A	AK6
PP2D1	TAF9
ZNF385D	RAD17
RARB	MARVELD2
LRRC3B	POC5
SCN11A	RASGRF2
CACNA1D	CKMT2
CHDH	VCAN
IL17RB	HAPLN1
ACTR8	NR2F1
SELENOK	FAM172A
CACNA2D3	POU5F2
ERC2	KIAA0825
FHIT	SLF1
SYNPR	MCTP1
TAFA1	PAM
TAFA4	GIN1
MDFIC2	PPIP5K2
ROBO2	MACIR
ROBO1	NUDT12
CADM2	TMEM232
NSUN3	SLC25A46
EPHA6	WDR36
ARL6	CAMK4
CRYBG3	STARD4
RIOX2	EPB41L4A

GABRR3	COMMD10
OR5AC2	SEMA6A
OR5H1	DTWD2
OR5H14	DMXL1
OR5H15	TNFAIP8
OR5H6	HSD17B4
OR5H2	SRFBP1
OR5K4	LOX
OR5K3	ZNF474
OR5K1	AC010255.3
OR5K2	SNCAIP
CLDND1	SNX2
DZIP3	SNX24
RETNLB	PPIC
TRAT1	CHSY3
GUCA1C	FSTL4
MORC1	NEUROG1
C3orf85	CXCL14
DPPA2	SLC25A48
DPPA4	IL9
LSAMP	PRELID2
IGSF11	GRXCR2
STXBP5L	SH3RF2
POLQ	TCERG1
ARGFX	GPR151
FBXO40	PPP2R2B
HCLS1	STK32A
GOLGB1	DPYSL3
IQCB1	GLRA1
EAF2	GRIA1
SLC15A2	MRPL22
ILDR1	KIF4B
CD86	TENM2
CASR	SLIT3
CSTA	SPDL1
CPNE4	DOCK2
EPHB1	INSYN2B
SOX14	FOXI1
RBP2	C5orf58
RBP1	LCP2
NMNAT3	KCNIP1
CLSTN2	KCNMB1
P2RY1	DRD1
ARHGEF26	SFXN1
DHX36	HRH2
GPR149	CPLX2
RSRC1	DUSP22

MLF1	IRF4
GFM1	EXOC2
LXN	HUS1B
RARRES1	FOXQ1
MFSD1	FOXF2
IQCJ-SCHIP1	BMP6
IQCJ	TXNDC5
KPNA4	BLOC1S5-TXNDC5
ARL14	BLOC1S5
PPM1L	EEF1E1-BLOC1S5
B3GALNT1	EEF1E1
NMD3	AL024498.2
SPTSSB	SYCP2L
OTOL1	ELOVL2
SI	SMIM13
SLITRK3	ERVFRD-1
BCHE	NEDD9
ZBBX	TMEM170B
SERPINI2	PHACTR1
WDR49	TBC1D7
PDCD10	CDKAL1
SERPINI1	TDP2
GOLIM4	ACOT13
MECOM	C6orf62
ACTRT3	H3C12
MYNN	H2AC17
LRRC34	H2BC17
LRRIQ4	OR2B2
SPATA16	DNAH8
NLGN1	UNC5CL
SOX2	OARD1
FGF12	TSPO2
MB21D2	APOBEC2
PLAAT1	NFYA
ATP13A5	TREML1
ATP13A4	TREM2
RAB28	TREML2
KCNIP4	TREML4
ARAP2	TREM1
GRXCR1	CD2AP
COX7B2	PKHD1
TECRL	FAM83B
CENPC	PRIM2
STAP1	IRAK1BP1
UBA6	PHIP
GNRHR	ELOVL4
TMPRSS11D	TTK

TMPRSS11A	BCKDHB
TMPRSS11F	CYB5R4
TMPRSS11B	MRAP2
YTHDC1	CEP162
TMPRSS11E	RNGTT
UGT2B17	PNISR
UGT2B15	USP45
UGT2B10	PRDM13
UGT2A3	HACE1
UGT2B7	KPNA5
UGT2B11	FAM162B
UGT2B28	GPRC6A
UGT2B4	TBC1D32
UGT2A1	GJA1
UGT2A2	HEY2
SULT1B1	NCOA7
AC108941.2	HINT3
SULT1E1	TRMT11
CSN1S1	NMBR
CSN2	GJE1
STATH	VTA1
HTN3	ADGB
HTN1	PPP1R14C
PRR27	IYD
ODAM	PLEKHG1
FDCSP	SYNE1
CSN3	MYCT1
CABS1	FBXO5
SMR3A	MTRF1L
SMR3B	RGS17
OPRPN	PACRG
MUC7	AC013470.2
AMTN	SCIN
GC	ARL4A
NPFFR2	ETV1
ADAMTS3	DGKB
PARM1	AGMO
NAA11	MACC1
GK2	ITGB8
RASGEF1B	DNAH11
MMRN1	CDCA7L
CCSER1	NFE2L3
GRID2	HNRNPA2B1
ATOH1	CBX3
UNC5C	SNX10
STPG2	HOXA1
CENPE	HOXA2

TACR3	HOXA3
UGT8	HOXA4
NDST4	HOXA5
FREM3	HOXA6
GYPE	HOXA7
GYPB	HOXA9
GYPA	AC004080.3
AC098588.2	HOXA10
TTC29	HOXA11
DCHS2	GHRHR
PLRG1	ADCYAP1R1
FGB	NPSR1
FGA	DPY19L1
FGG	ANLN
LRAT	STARD3NL
RBM46	AMPH
FSTL5	VPS41
NAF1	POU6F2
NPY1R	GLI3
NPY5R	ABCA13
TKTL2	SEMA3E
TMA16	SEMA3A
MARCHF1	SEMA3D
CPE	STEAP1
GLRA3	STEAP2
GPM6A	CFAP69
STOX2	FAM237B
ENPP6	GTPBP10
FRG1	CLDN12
CTNND2	HEPACAM2
GHR	VPS50
FGF10	GNGT1
MRPS30	TFPI2
DEPDC1B	GNG11
ELOVL7	BET1
ERCC8	COL1A2
KIF2A	CASD1
IPO11	SGCE
LRRC70	PEG10
HTR1A	PPP1R9A
RNF180	ORC5
RGS7BP	IMMP2L
MAST4	DOCK4
SV2C	GPR85
IQGAP2	SMIM30
RASGRF2	PPP1R3A
COX7C	FOXP2

TMEM161B	MDFIC
ADGRV1	TFEC
MCTP1	CFTR
FAM174A	ASZ1
ST8SIA4	KCND2
SLCO4C1	TSPAN12
SLCO6A1	PLXNA4
TSLP	CNTNAP2
EPB41L4A	PTPRN2
KCNN2	VIPR2
TRIM36	ERICH1
PGGT1B	DLGAP2
SEMA6A	DEFA5
DTWD2	MSRA
HSD17B4	PRSS51
FAM170A	PRSS55
FTMT	RP1L1
SLC27A6	C8orf74
ISOC1	SOX7
ADAMTS19	PINX1
MINAR2	PINX1
SMIM32	XKR6
TRPC7	LONRF1
PPP2R2B	TRMT9B
STK32A	DLC1
JAKMIP2	C8orf48
SPINK1	GFRA2
SCGB3A2	NEFM
C5orf46	NEFL
SPINK5	DOCK5
SPINK14	GNRH1
SPINK6	KCTD9
MARCOL	CDCA2
SPINK13	ADRA1A
SPINK7	STMN4
SPINK9	TRIM35
FBXO38	PTK2B
HTR4	CHRNA2
GLRA1	EPHX2
NMUR2	CLU
GRIA1	SCARA3
ATP10B	PURG
GABRB2	WRN
GABRA6	NRG1
GABRA1	TTI2
GABRG2	MAK16
KCNIP1	RNF122

GABRP	DUSP26
RANBP17	ZMAT4
TLX3	RPS20
NSG2	MOS
MSX2	PLAG1
SLC35B3	CHCHD7
TFAP2A	IMPAD1
GCNT2	ARMC1
C6orf52	MTFR1
PAK1IP1	CPA6
TMEM14C	PREX2
TMEM14B	C8orf34
AL024498.2	SULF1
MAK	SLCO5A1
GCM2	PRDM14
SYCP2L	NCOA2
PHACTR1	TRAM1
ID4	TERF1
MBOAT1	SBSPON
E2F3	C8orf89
NRSN1	IMPA1
DCDC2	SLC10A5
KAAG1	ZFAND1
MRS2	CHMP4C
GPLD1	SNX16
ALDH5A1	MMP16
KIAA0319	NECAB1
TDP2	C8orf88
OR2B6	PIP4P2
ZNF165	OTUD6B
ZSCAN16	LRRC69
ZKSCAN8	SLC26A7
ZSCAN9	RUNX1T1
ZKSCAN4	TRIQK
NKAPL	RIMS2
ZSCAN26	DPYS
AL021997.3	DCSTAMP
PGBD1	LRP12
ZSCAN31	ZFPM2
ZKSCAN3	OXR1
ZSCAN12	ABRA
ZSCAN23	ANGPT1
GPX6	RSPO2
GPX5	EIF3E
ZBED9	EMC2
TRIM27	AARD
ZNF311	SLC30A8

OR2W1	MAL2
OR2B3	CCN3
OR2J1	EFR3A
OR2J3	TMEM71
OR2J2	ZFAT
OR14J1	KCNK9
OR5V1	TRAPPC9
OR12D3	TSNARE1
OR12D2	ADGRB1
OR11A1	ARC
OR10C1	DMRT1
OR2H1	SMARCA2
MAS1L	RFX3
OR2I1P	TTC39B
UBD	SNAPC3
GABBR1	PSIP1
OR2H2	CCDC171
MOG	CNTLN
ZFP57	SH3GL2
HLA-F	ADAMTSL1
DNAH8	TUSC1
GLP1R	CAAP1
SAYSD1	PLAA
KCNK5	IFT74
KCNK17	LRRC19
KCNK16	TEK
KIF6	EQTN
DAAM2	MOB3B
MOCS1	CEMIP2
LRFN2	ABHD17B
CD2AP	C9orf85
ADGRF2	C9orf57
ADGRF4	ZFAND5
OPN5	TMC1
PTCHD4	ALDH1A1
MMUT	ANXA1
CENPQ	VPS13A
GLYATL3	GNA14
C6orf141	CEP78
RHAG	PSAT1
CRISP2	AGTPBP1
CRISP3	CKS2
PGK2	SECISBP2
CRISP1	SEMA4D
DEFB133	GADD45G
DEFB114	ROR2
DEFB113	SPTLC1

DEFB110	FGD3
DEFB112	SUSD3
TFAP2D	CARD19
TFAP2B	NINJ1
PKHD1	WNK2
HCRTR2	PHF2
GFRAL	BARX1
HMGCLL1	GABBR2
BMP5	ANKS6
ADGRB3	CACNA1B
HTR1B	NLGN4X
MEI4	GLRA2
HTR1E	FANCB
CGA	MOSPD2
EPHA7	ASB9
MANEA	ASB11
FUT9	PIGA
UFL1	VEGFD
FHL5	PIR
GPR63	BMX
NDUFAF4	ACE2
KLHL32	AC097625.2
MMS22L	CLTRN
POU3F2	CA5B
FBXL4	ZRSR2
FAXC	AP1S2
COQ3	NHS
GRIK2	SCML1
HACE1	SCML2
FRK	MAGEB16
GPRC6A	CCNB3
RFX6	DGKK
VGLL2	NUDT10
ROS1	EZHIP
AL132671.2	NUDT11
DCBLD1	TRO
GOPC	PFKFB1
NUS1	APEX2
SLC35F1	ALAS2
HSF2	PAGE2B
SERINC1	FAM104B
PKIB	MTRNR2L10
FABP7	RRAGB
SMPDL3A	UBQLN2
CLVS2	ZXDA
TRDN	AMER1
NKAIN2	ASB12

GRM1
RAB32
ADGB
VIP
OPRM1
IPCEF1
AL357075.4
LPA
PLG
MAP3K4
AGPAT4
PRKN
PACRG
C6orf118
PDE10A
TBXT
PRR18
SFT2D1
MPC1
RPS6KA2
RAMACL
AL159163.1
RNASET2
FGFR1OP
CCR6
TCP10L2
GPR31
UNC93A
TTLL2
TCP10
AFDN
KIF25
FRMD1
DACT2
SMOC2
ICA1
NXPH1
NDUFA4
PHF14
THSD7A
TMEM106B
VWDE
AC013470.2
TWISTNB
TMEM196
ABCB5
SP8
KIF4A
GDPD2
DLG3
RADX
RNF128
PAK3
ALG13
TRPC5
TRPC5OS
ARHGAP36
FMR1
FMR1NB

SP4
DNAH11
C7orf31
NPVF
NEUROD6
ITPRID1
PPP1R17
PDE1C
NPSR1
TBX20
ANLN
AOAH
ELMO1
GPR141
EPDR1
NME8
SFRP4
STARD3NL
POU6F2
ABCA13
VWC2
ZPBP
VSTM2A
PCLO
SEMA3E
GRM3
KIAA1324L
DMTF1
TMEM243
CROT
ABCB4
ABCB1
RUNDC3B
SLC25A40
DBF4
ADAM22
SRI
STEAP4
ZNF804B
TEX47
VPS50
CALCR
GNGT1
PPP1R9A
PON1
PON3
PON2

ASB4
PDK4
DYNC1I1
SLC25A13
SEM1
DLX6
DLX5
SDHAF3
TAC1
ASNS
RELN
IMMP2L
DOCK4
CFTR
ASZ1
CTTNBP2
LSM8
ANKRD7
PTPRZ1
AASS
FEZF1
CADPS2
RNF133
RNF148
TAS2R16
SLC13A1
IQUB
NDUFA5
ASB15
LMOD2
WASL
HYAL4
SPAM1
TMEM229A
SSU72P8
GPR37
C7orf77
POT1
GRM8
TMEM178B
AGK
DENND11
WEE2
SSBP1
TAS2R3
TAS2R4
TAS2R5

PRSS37
MGAM
OR9A4
CLEC5A
TAS2R38
MGAM2
PRSS58
MTRNR2L6
PRSS1
PRSS2
EPHB6
TRPV6
TRPV5
LLCFC1
KEL
OR9A2
OR6V1
PIP
TAS2R39
TAS2R40
GSTK1
TMEM139
CASP2
CLCN1
FAM131B
ZYX
EPHA1
OR2F1
OR6B1
OR2A5
OR2A25
OR2A12
OR2A2
OR2A14
ARHGEF5
NOBOX
TPK1
CNTNAP2
ZNF467
ZNF862
ATP6V0E2
ACTR3C
ZBED6CL
LRRC61
RARRES2
REPIN1
ZNF775

AC073111.4
GIMAP8
GIMAP7
GIMAP4
GIMAP6
GIMAP2
DPP6
SHH
PTPRN2
CSMD1
SGCZ
ADAM28
ADAMDEC1
ADAM7
ADRA1A
PURG
NRG1
ADAM18
ADAM2
IDO1
IDO2
TCIM
ZMAT4
ST18
ALKAL1
RB1CC1
NPBWR1
OPRK1
RP1
XKR4
TMEM68
TGS1
LYN
SDR16C5
CA8
NKAIN3
GGH
TTPA
YTHDF3
BHLHE22
CYP7B1
PREX2
C8orf34
TRAM1
LACTB2
XKR9
EYA1

MSC
TRPA1
KCNB2
TERF1
CRISPLD1
HNF4G
FABP12
RALYL
LRRCC1
E2F5
RBIS
CA13
CA1
CA3
CA2
WWP1
RMDN1
CPNE3
CNGB3
CNBD1
TRIQK
TMEM74
TRHR
NUDCD1
ENY2
PKHD1L1
EBAG9
SYBU
KCNV1
CSMD3
OC90
AC100868.1
HHLA1
KCNQ3
LRRC6
TMEM71
FAM135B
COL22A1
KCNK9
DMRT1
DMRT3
DMRT2
DMAC1
PTPRD
IZUMO3
MOB3B
IFNK

C9orf72
LINGO2
PGM5
TMEM252
PIP5K1B
FAM122A
PRKACG
FXN
AL358113.1
TRPM3
C9orf57
GDA
ANXA1
RORB
TRPM6
C9orf40
CARNMT1
NMRK1
OSTF1
PCSK5
RFK
GNA14
CEP78
RASEF
FRMD3
IDNK
UBQLN1
GKAP1
KIF27
C9orf64
HNRNPK
RMI1
SLC28A3
NTRK2
AGTPBP1
SEMA4D
DIRAS2
SYK
PLPPR1
BAAT
MRPL50
ZNF189
ALDOB
PGAP4
RNF20
GRIN3A
PPP3R2

CYLC2
SMC2
OR13F1
OR13C4
OR13C3
OR13C8
OR13C5
OR13C2
OR13C9
OR13D1
ASTN2
AL160272.2
TLR4
BRINP1
CDK5RAP2
SCML1
RAI2
BEND2
FAM47A
KLF8
